# Supplementary material for: Prion-induced ferroptosis is facilitated by RAC3
Source: Nat Commun. 2025 Jun 25;16:5385. doi: 10.1038/s41467-025-60793-3 (PMC12198409; doi:10.1038/s41467-025-60793-3)
Supplement: Supplementary file 1 — Supplementary Information [file 41467_2025_60793_MOESM1_ESM.pdf]

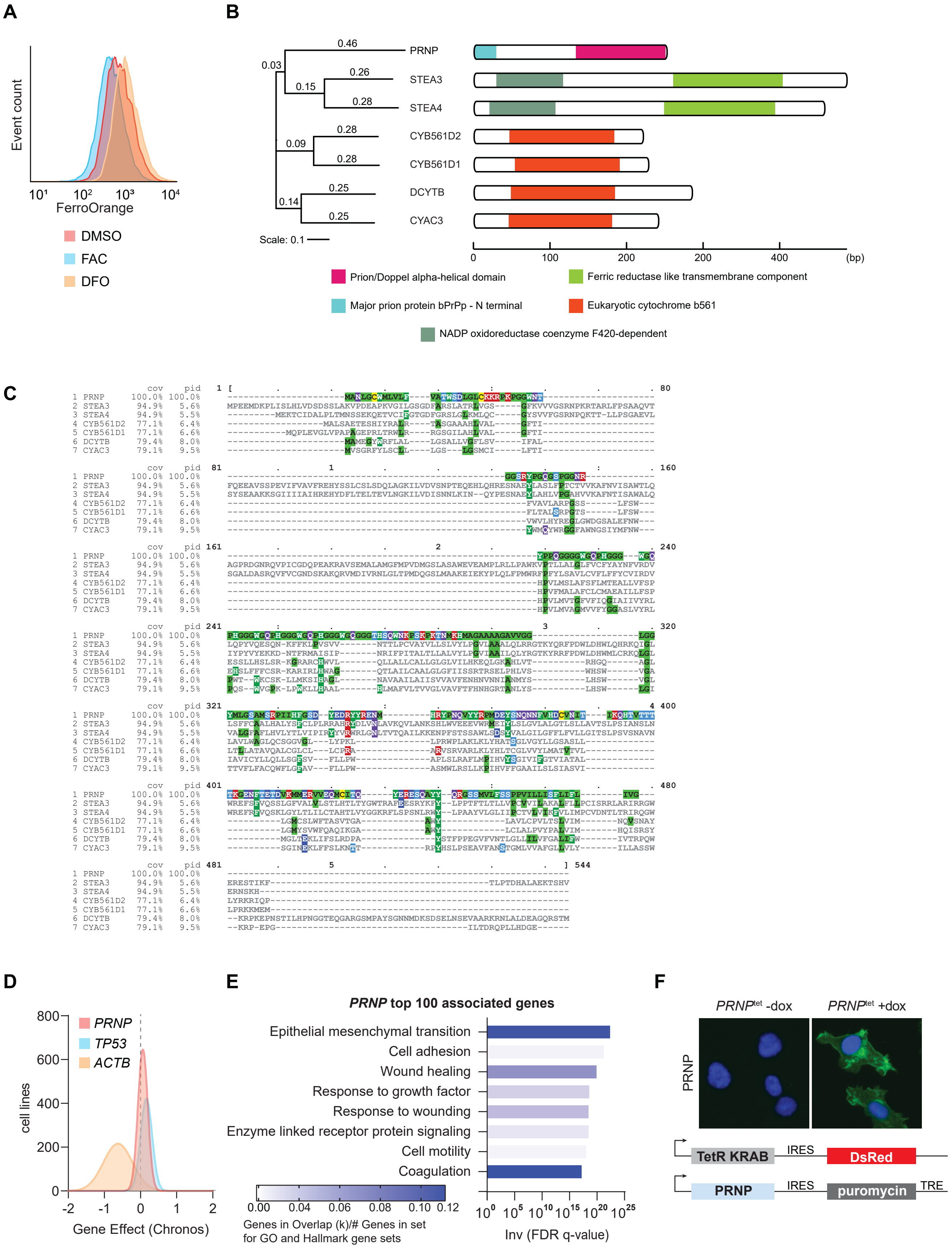
A
Event count
10¹ 10² 10³ 10⁴
FerroOrange
DMSO
FAC
DFO
B
0.46
PRNP
0.03
0.26
STEA3
0.15
0.28
STEA4
0.28
CYB561D2
0.09
0.28
CYB561D1
0.25
DCYTB
0.14
0.25
CYAC3
Scale: 0.1
0 100 200 200 400 (bp)
Prion/Doppel alpha-helical domain
Ferric reductase like transmembrane component
Major prion protein bPrPp - N terminal
Eukaryotic cytochrome b561
NADP oxidoreductase coenzyme F420-dependent
C
D
800
600
400
200
0
cell lines
PRNP
TP53
ACTB
-2 -1 0 1 2
Gene Effect (Chronos)
E
PRNP top 100 associated genes
Epithelial mesenchymal transition
Cell adhesion
Wound healing
Response to growth factor
Response to wounding
Enzyme linked receptor protein signaling
Cell motility
Coagulation
0.00 0.04 0.06 0.08 0.10 0.12
Genes in Overlap (k)/# Genes in set
for GO and Hallmark gene sets
10⁰ 10⁵ 10¹⁰ 10¹⁵ 10²⁰ 10²⁵
Inv (FDR q-value)
F
PRNPtet -dox
PRNPtet +dox
PRNP
TetR KRAB
IRES
DsRed
PRNP
IRES
puromycin
TRE

Supp Figure 1 Characterization of PrP^C function through iron response, domain analysis, and genetic screens

A Control data corresponding to Fig. 1A demonstrating FerroOrange response to iron stimuli.

B-C Pfam domain and cluster analysis of PRNP and classic ferroreductases.

D Histogram showing the growth distribution of cell lines following whole-genome CRISPR knockout screens in 1100 cell lines. Knockout cells of both tumor suppressor P53 (*TP53*) and *PRNP* show increased growth kinetics, while knockout of beta-actin (*ACTB*) is detrimental to cell growth.

E Gene Ontology (GO) and Hallmark gene set associations of 100 most highly associated genes with PrP^C expression (encoded by the gene *PRNP*). Color intensity indicates fraction of genes found in the gene set divided by total number of genes in a given set. FDR, false discovery rate.

F Creation of a tetracycline (doxycycline, dox) inducible PrP^C HT-1080 cell line. Initially, PrP^C expression is suppressed by TetR KRAB binding to the downstream Tetracycline Responsive Element (TRE) as detected by antibodies against the prion protein (left image). Addition of doxycycline reveals robust cellular PrP^C (right image) and puromycin resistance after three days of treatment.

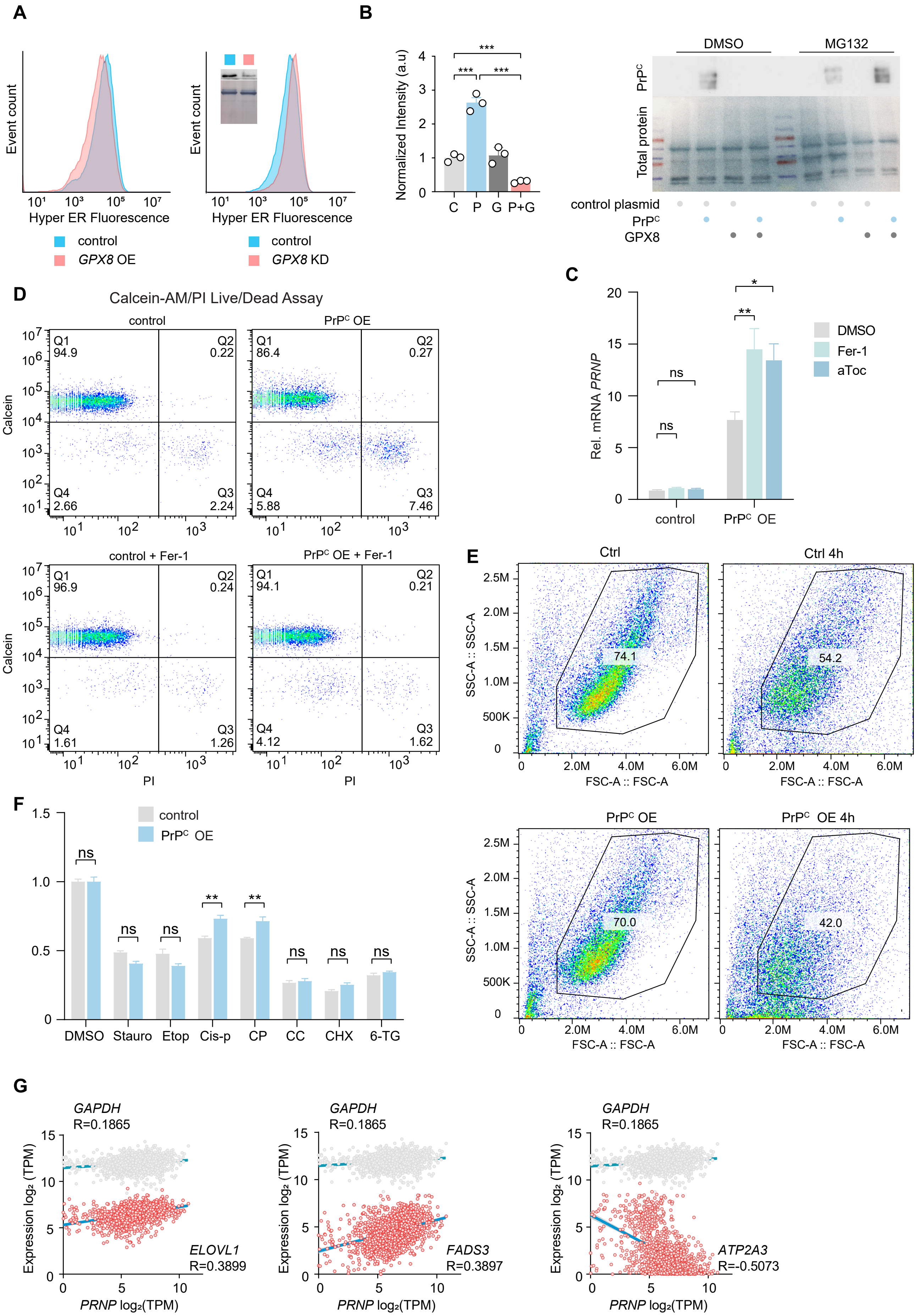
A
Event count
Hyper ER Fluorescence
control
GPX8 OE
GPX8 KD
B
Normalized Intensity (a.u)
C P G P+G
DMSO
MG132
PrPC
Total protein
control plasmid
GPX8
C
Rel. mRNA PRNP
DMSO
Fer-1
aToc
control
PrPC OE
D
Calcein-AM/PI Live/Dead Assay
control
PrPC OE
control + Fer-1
PrPC OE + Fer-1
Calcein
PI
E
Ctrl
Ctrl 4h
PrPC OE
PrPC OE 4h
SSC-A :: SSC-A
FSC-A :: FSC-A
F
DMSO Stauro Etop Cis-p CP CC CHX 6-TG
G
GAPDH
R=0.1865
ELOVL1
R=0.3899
FADS3
R=0.3897
ATP2A3
R=-0.5073
Expression log2 (TPM)
PRNP log2(TPM)

Supp Figure 2 GPX8 and PrP^C modulate ER stress, surface expression, and ferroptosis sensitivity

A The effects of GPX8 knockdown (KD) and overexpression (OE) on Hyper ER fluorescence intensity in HT-1080 cells

B (left) Total surface expression of PrP^C detected by flow cytometry. (Right) Western detection of PrP^C in DMSO or MG132-treated HEK293 cells following transfection. C, Control vector; P, PrP^C (*PRNP* OE); G, *GPX8* OE; P+G, PrP^C + GPX8.

C Relative expression of *PRNP* in PrP^C OE and control cells compared to Fer-1 treatment or DMSO treated condition as well as aToc treatment detected by qPCR.

D Dot plot displaying four quadrant gates recorded by flow cytometry analysis for cells stained with calcein AM and propidium iodide (PI).

E Dot plot corresponding to Fig. 2C demonstrating cell state after ferroptotic inducer treatment over time.

F Survival of PrP^C OE and control cells treated with equivalent death-inducing concentrations of different pharmacological agents.

G Pearson correlation analysis of *PRNP* mRNA expression with *FADS3*, *ELOVL1*, and *ATP2A3* among 18,575 genes determined in 1,393 individual cell lines with shown R values. *GAPDH* is included as a reference gene. TPM, transcripts per million.

Significance was determined by two-tailed t-test (B) or two-way ANOVA multiple comparisons with Tukey post-test (C and F). $^*P < 0.05$, $^{**}P < 0.01$, $^{***}P < 0.001$, $^{****}P < 0.0001$.

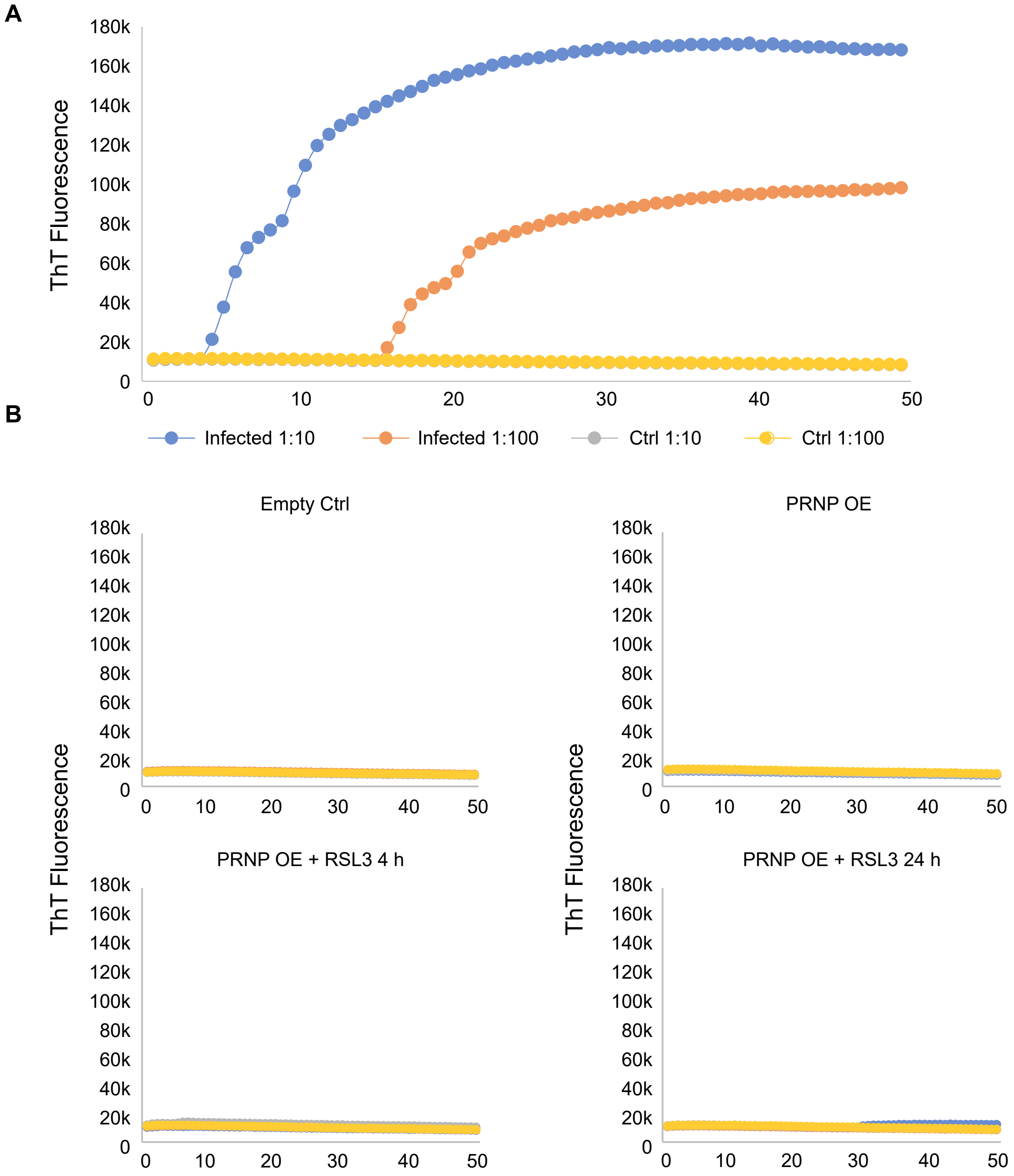

A
ThT Fluorescence
180k
160k
140k
120k
100k
80k
60k
40k
20k
0
0
10
20
30
40
50
Infected 1:10
Infected 1:100
Ctrl 1:10
Ctrl 1:100
B
Empty Ctrl
PRNP OE
PRNP OE + RSL3 4 h
PRNP OE + RSL3 24 h
ThT Fluorescence
ThT Fluorescence

Supp Figure 3 RT-QuIC detection of seeding activity and absence of pathogenic signal in cellular lysates

A RT-QuiC assay reveals PrP^{Sc} seeding following addition of pathogenic protein lysates. Samples were run as 4 replicate reactions. The curve is displayed as the average fluorescence from the 4 replicate wells.

B Cell lysates from the respective conditions done in parallel with (A) reveal absence of pathogenic PrP signal in overexpressing cell lines and those induced for ferroptosis. Four samples from each condition were tested in 8 replicate reactions. The curves are displayed as average fluorescence from the 8 replicate wells.

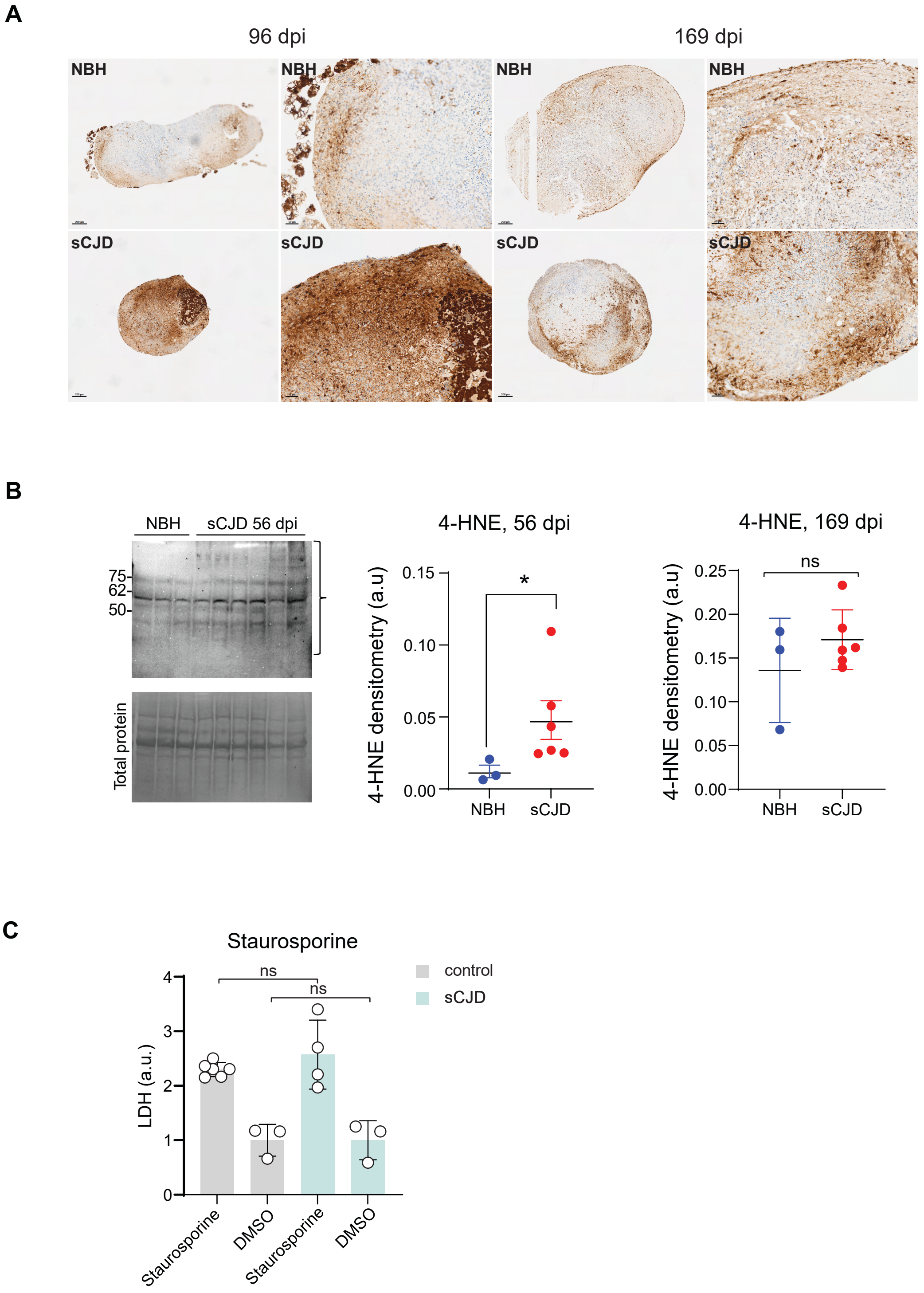
A
96 dpi
169 dpi
NBH
NBH
NBH
NBH
sCJD
sCJD
sCJD
sCJD
B
NBH
sCJD 56 dpi
75
62
50
Total protein
4-HNE, 56 dpi
4-HNE densitometry (a.u)
0.15
0.10
0.05
0.00
*
NBH
sCJD
4-HNE, 169 dpi
4-HNE densitometry (a.u)
0.25
0.20
0.15
0.10
0.05
0.00
ns
NBH
sCJD
C
Staurosporine
LDH (a.u.)
4
3
2
1
0
ns
ns
control
sCJD
Staurosporine
DMSO
Staurosporine
DMSO

Supp Figure 4 FABP5, HNE markers, and LDH release in cerebral organoids following sCJD infection

A Cerebral organoids at 96 and 169 days postinfection (dpi) with normal brain homogenate (NBH) or sCJD homogenates with immunohistochemistry directed against FABP5.

B 4-HNE detection and quantification of cerebral organoids at 56 and 169 dpi with NBH or sCJD brain homogenates. The bracket indicates the region used for quantification, normalized to total protein. Significance was determined by Mann-Whitney test.

C LDH release from control (NBH) and sCJD infected organoids treated for 72h with 1 µM staurosporine or DMSO carrier.

Significance was determined by two-tailed t-test, ns, no significance.

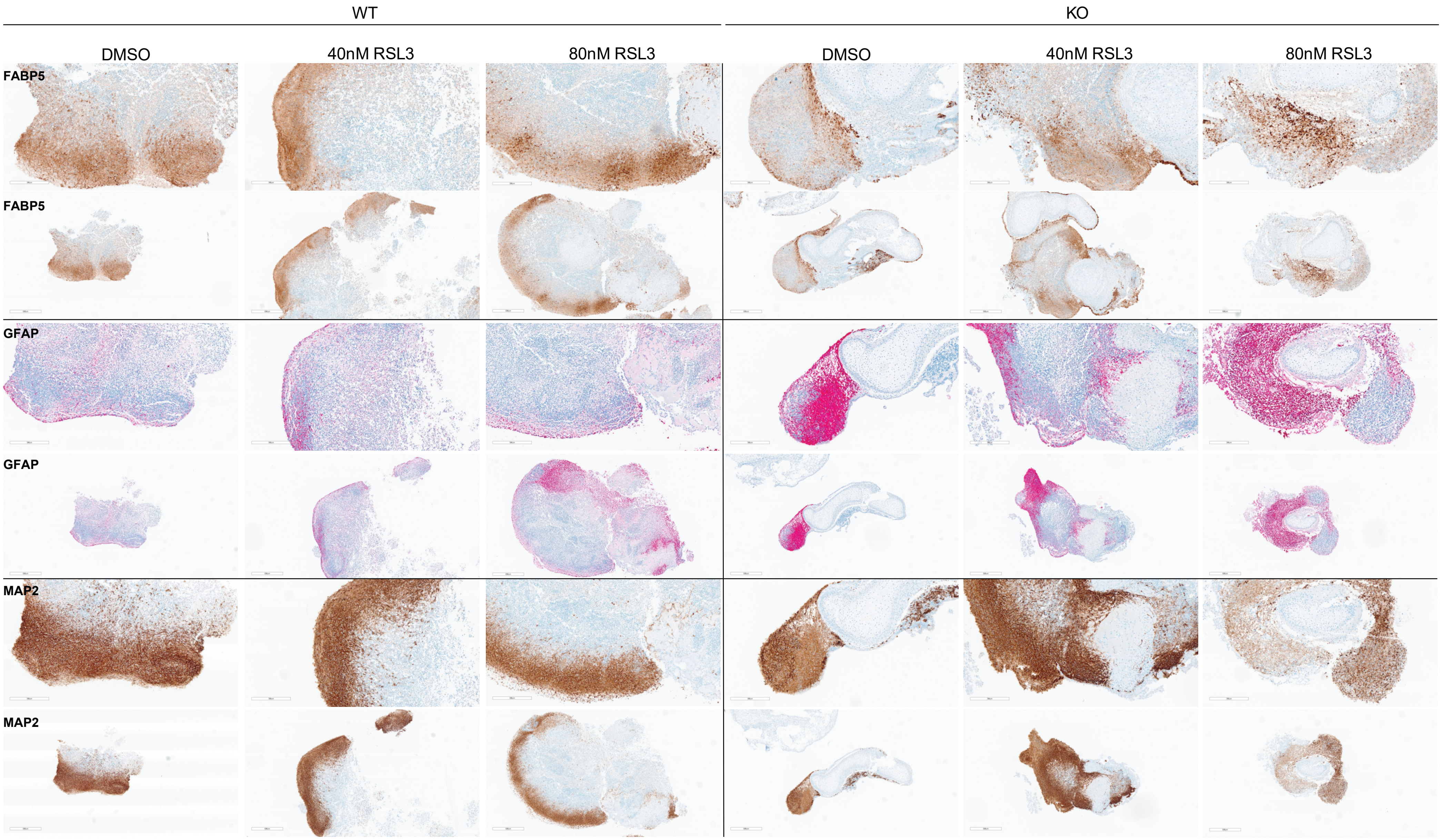
WT
KO
DMSO
40nM RSL3
80nM RSL3
DMSO
40nM RSL3
80nM RSL3
FABP5
FABP5
GFAP
GFAP
MAP2
MAP2

Supp Figure 5 Glial, ferroptosis and neuronal markers in *PRNP* knockout and control organoids

FABP5 (ferroptosis marker), GFAP (astrocyte marker) and MAP2 (neuronal marker) staining of prion gene (*PRNP*) WT and knock-out organoids that were grown to ~4 months old and treated with RSL3 for 48h.

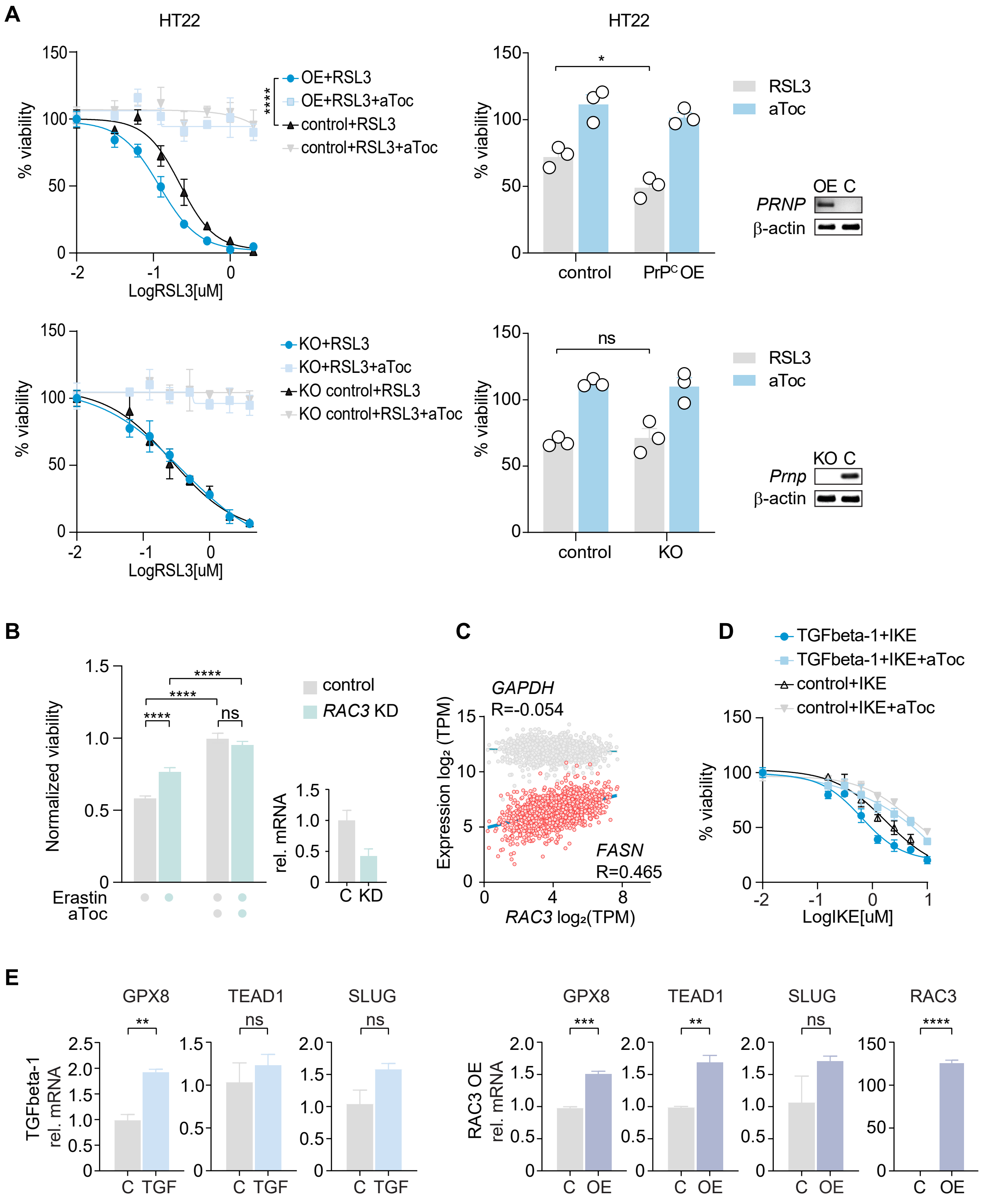
A
HT22
OE+RSL3
OE+RSL3+aToc
control+RSL3
control+RSL3+aToc
% viability
LogRSL3[uM]
RSL3
aToc
control
PrP^C OE
PRNP
β-actin
KO+RSL3
KO+RSL3+aToc
KO control+RSL3
KO control+RSL3+aToc
KO
Prnp
B
Normalized viability
Erastin
aToc
control
RAC3 KD
rel. mRNA
C
GAPDH
R=-0.054
FASN
R=0.465
Expression log2 (TPM)
RAC3 log2(TPM)
D
TGFbeta-1+IKE
TGFbeta-1+IKE+aToc
control+IKE
control+IKE+aToc
LogIKE[uM]
E
GPX8
TEAD1
SLUG
RAC3
TGFbeta-1 rel. mRNA
RAC3 OE rel. mRNA

Supp Figure 6 Dose-response, viability analyses and mesenchymal gene expression levels in cell models

A RSL3 Dose-response curves elucidate the sensitivity of human *PRNP* overexpression and *mPrnp* KO HT22 cells compared to empty vector control over 16-hours. Bar graph demonstrating cell viability under various conditions following treatment with 125 nM RSL3. Inset displays genomic PCR results for *β-actin*, *PRNP*, and murine *Prnp*.

B Survival of *RAC3* KD HT-1080 cells treated with Erastin rescued by 10 μM α-tocopherol (aToc). Inset shows relative *RAC3* expression level.

C Pearson correlation analysis of *FASN* mRNA expression with *RAC3* mRNA expression (R=0.465; $P < 0.0001$) over 18,575 genes determined for 1,393 individual cell lines. *GAPDH* is included as a reference gene. TPM, transcripts per million.

D Dose-response curves illustrate the sensitivity of TGFbeta-1 treated HT-1080 cells compared to DMSO treated control to IKE treatment over 16-hours.

E Relative expression of mesenchymal markers in *RAC3* OE and TGFbeta-1 treated cells (3 days) compared to empty vector or DMSO treated control detected by qPCR.

Significance was determined by two-tailed t-test (Bar graph A and E) or two-way ANOVA multiple comparisons with Tukey post-test (A, B and D). *P < 0.05, **P < 0.01, ***P < 0.001, ****P < 0.0001.

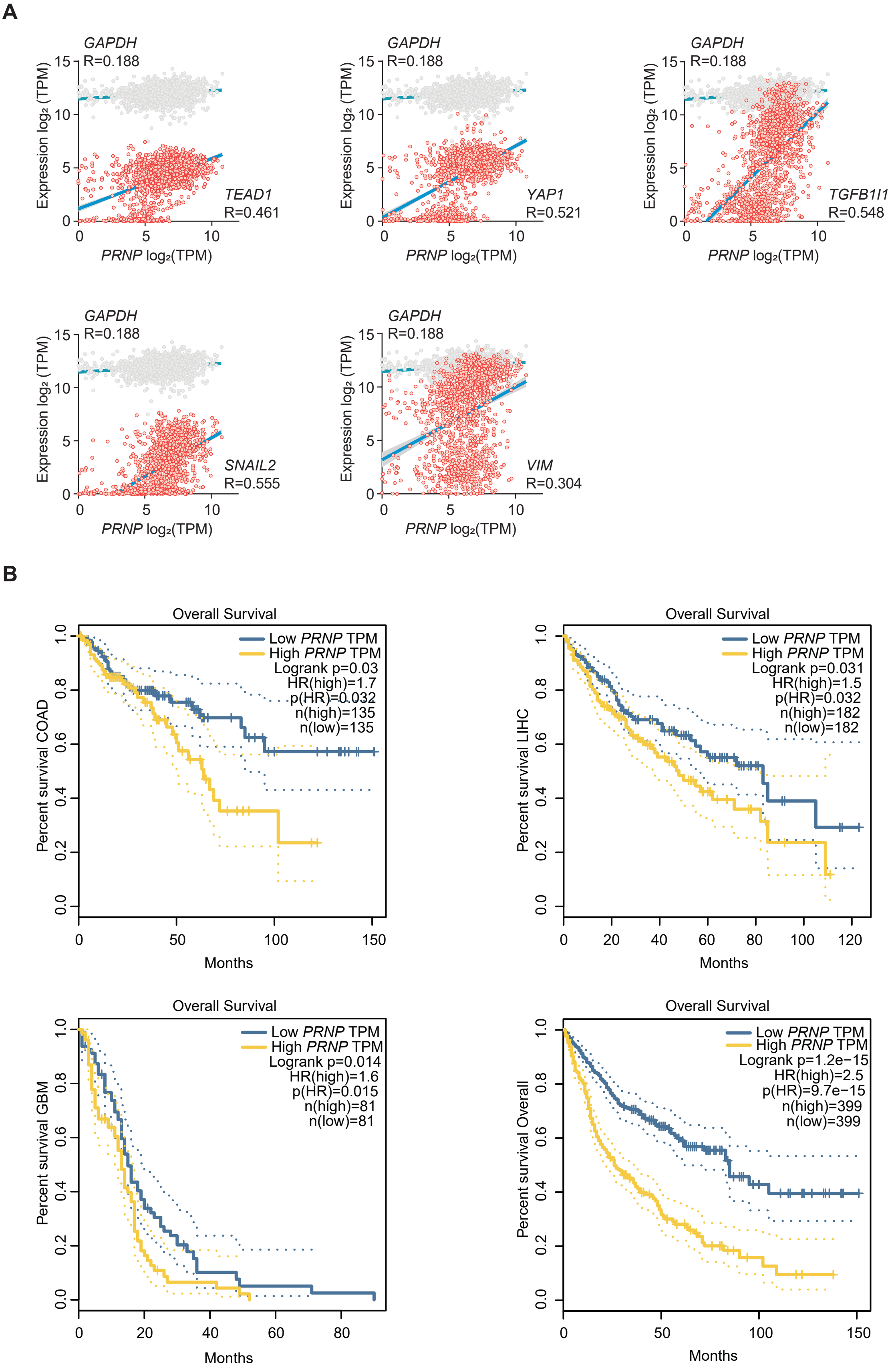
A
GAPDH
R=0.188
TEAD1
R=0.461
YAP1
R=0.521
TGFB1I1
R=0.548
SNAIL2
R=0.555
VIM
R=0.304
Expression log2 (TPM)
PRNP log2(TPM)
B
Overall Survival
Low PRNP TPM
High PRNP TPM
Logrank p=0.03
HR(high)=1.7
p(HR)=0.032
n(high)=135
n(low)=135
Percent survival COAD
Logrank p=0.031
HR(high)=1.5
p(HR)=0.032
n(high)=182
n(low)=182
Percent survival LIHC
Logrank p=0.014
HR(high)=1.6
p(HR)=0.015
n(high)=81
n(low)=81
Percent survival GBM
Logrank p=1.2e−15
HR(high)=2.5
p(HR)=9.7e−15
n(high)=399
n(low)=399
Percent survival Overall
Months

Supp Figure 7 Correlation analysis and survival data related to *PRNP* expression in various tumor types

A Pearson correlation analysis of epithelial and mesenchymal markers mRNA expression with *PRNP* mRNA expression (TEAD1, R=0.461, $P < 0.0001$; YAP1, R=0.521, $P < 0.0001$; TGFB1l1, R=0.548, $P < 0.0001$; SNAIL2, R=0.555, $P < 0.0001$; VIM, R=0.304, $P < 0.0001$) over 18,575 genes determined for 1,393 individual cell lines. *GAPDH* is included as a reference gene. TPM, transcripts per million.

B Kaplan-Meier curves of high *PRNP* (yellow) and low *PRNP* (dark blue) expression in given tumor tissues. Kaplan-Meier survival curves of patients divided by median gene expression amongst target genes in given tumor types. The calculated hazards ratio based on Cox PH Model. 95% CI as dotted line was added. The horizontal axis (x-axis) represents survival time in months, and the vertical axis (y-axis) shows percent patient survival. (Colon adenocarcinoma, COAD; Liver hepatocellular carcinoma, LIHC; glioblastoma multiforme, GBM).

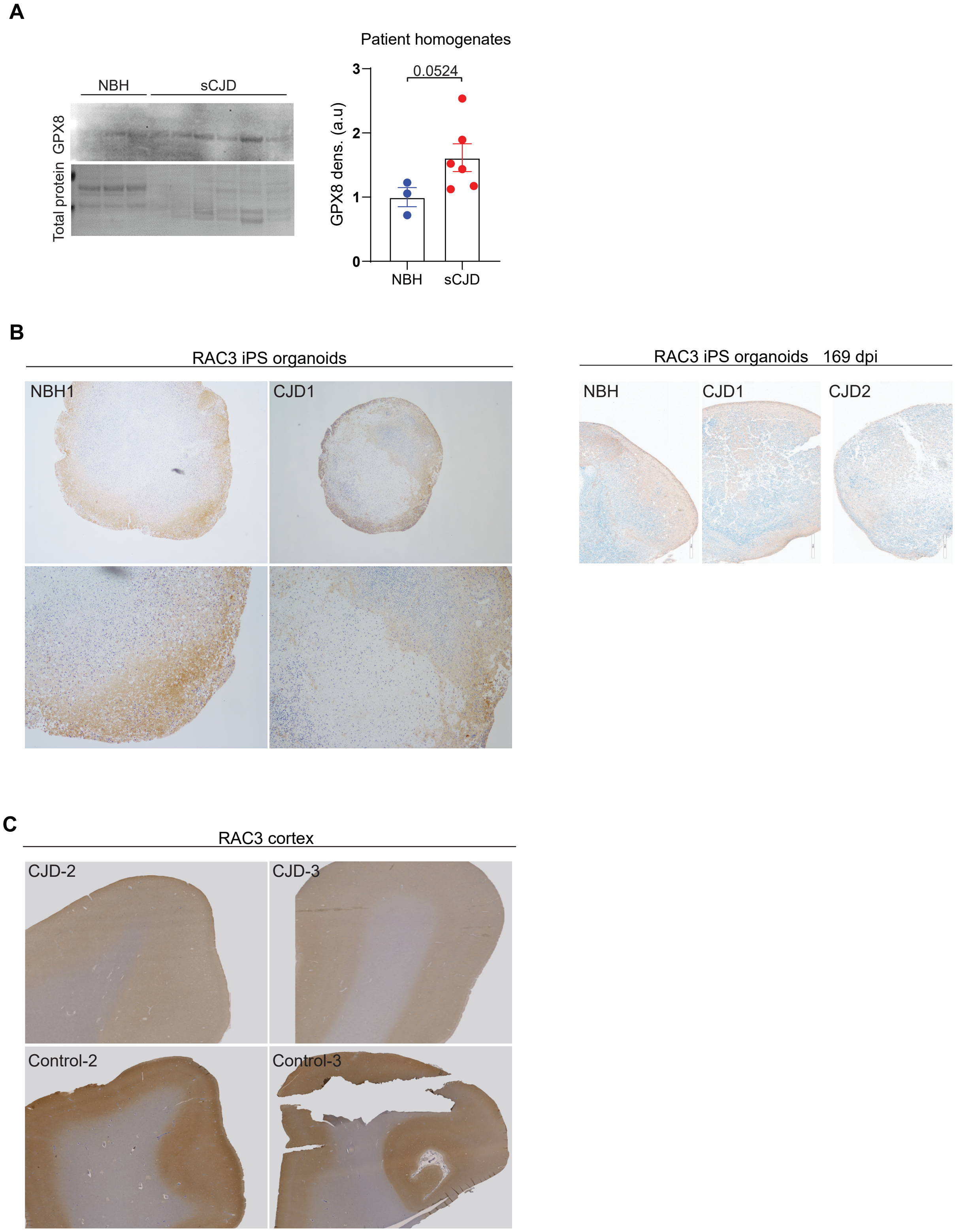
A
Patient homogenates
NBH
sCJD
GPX8
Total protein
0.0524
GPX8 dens. (a.u)
3
2
1
0
NBH
sCJD
B
RAC3 iPS organoids
NBH1
CJD1
RAC3 iPS organoids 169 dpi
NBH
CJD1
CJD2
C
RAC3 cortex
CJD-2
CJD-3
Control-2
Control-3

Supp Figure 8 Protein expression and RAC3 analysis in brain tissue from sporadic CJD and control cases

A Western blotting and densitometry comparing GPX8 protein levels in the brain tissue of people who died from sporadic CJD with tissue from patients who died of a non-brain-related condition.

B RAC3 staining intensity at 169dpi in iPS-derived brain organoids mock-infected with normal brain homogenates (NBH) compared to infection with two sCJD brain homogenates (CJD1, CJD2).

C Immunohistochemical detection of RAC3 in the superior frontal gyrus in control cases (Control-2 and Control-3, bottom panel) and cases with Creutzfeldt-Jakob disease (CJD-2 and CJD-3, upper panel). RAC3 is strongly expressed in the neocortical neuropil of the control cases but markedly reduced in the sCJD cases.

Significance was determined by two-tailed Welch's t-test.

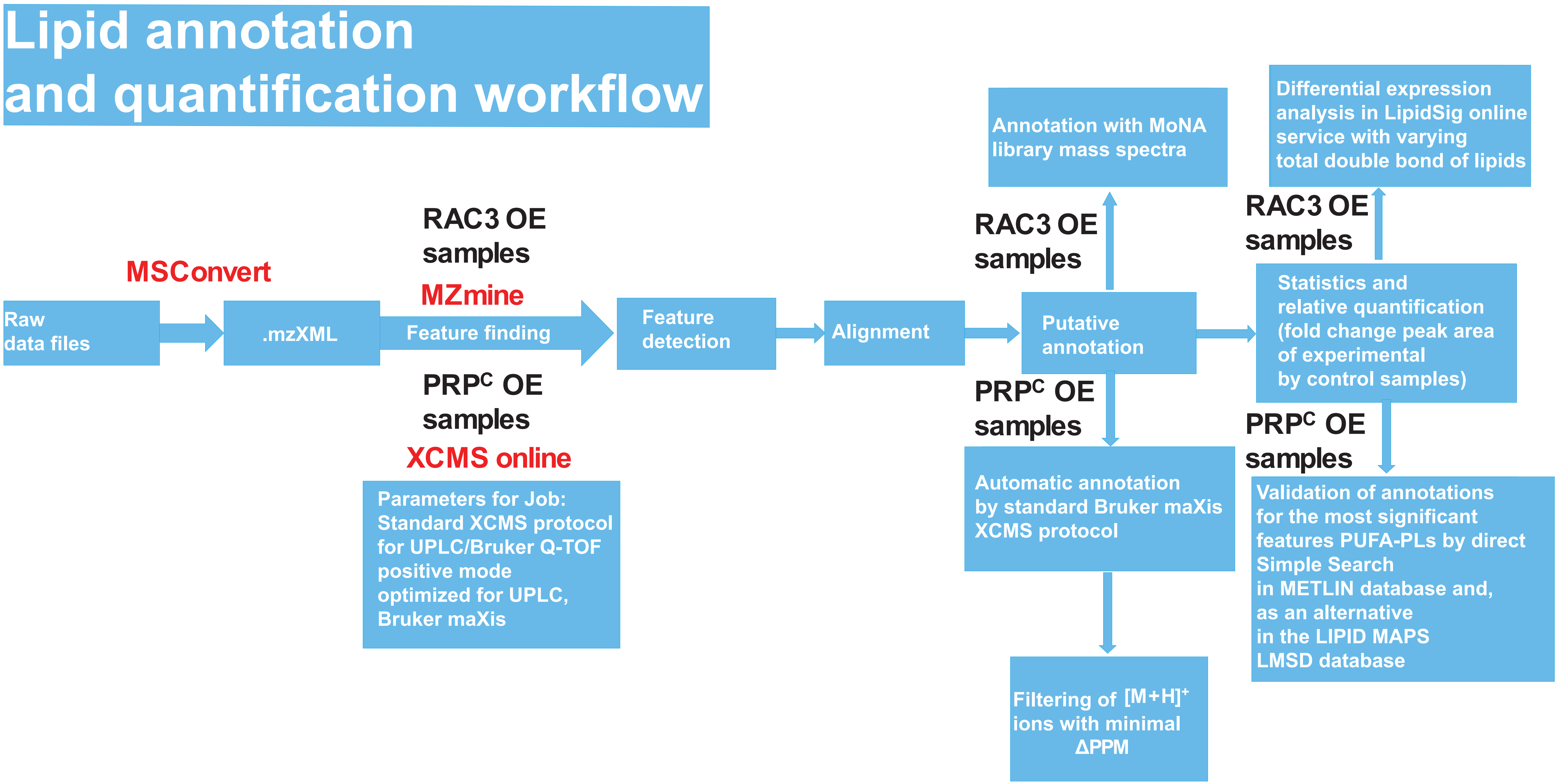
Lipid annotation
and quantification workflow
Raw
data files
MSConvert
.mzXML
RAC3 OE
samples
MZmine
Feature finding
PRP^{C} OE
samples
XCMS online
Parameters for Job:
Standard XCMS protocol
for UPLC/Bruker Q-TOF
positive mode
optimized for UPLC,
Bruker maXis
Feature
detection
Alignment
Annotation with MoNA
library mass spectra
RAC3 OE
samples
Putative
annotation
PRP^{C} OE
samples
Automatic annotation
by standard Bruker maXis
XCMS protocol
Filtering of [M+H]$^{+}$
ions with minimal
ΔPPM
Differential expression
analysis in LipidSig online
service with varying
total double bond of lipids
RAC3 OE
samples
Statistics and
relative quantification
(fold change peak area
of experimental
by control samples)
PRP^{C} OE
samples
Validation of annotations
for the most significant
features PUFA-PLs by direct
Simple Search
in METLIN database and,
as an alternative
in the LIPID MAPS
LMSD database

Supp Figure 9 Lipidomics analyses in HT1080 overexpression cell lines

A Schematic overview of lipidomics analyses in RAC3 OE and PrP^{C} OE HT1080 cells.

Sup Fig 6a actin

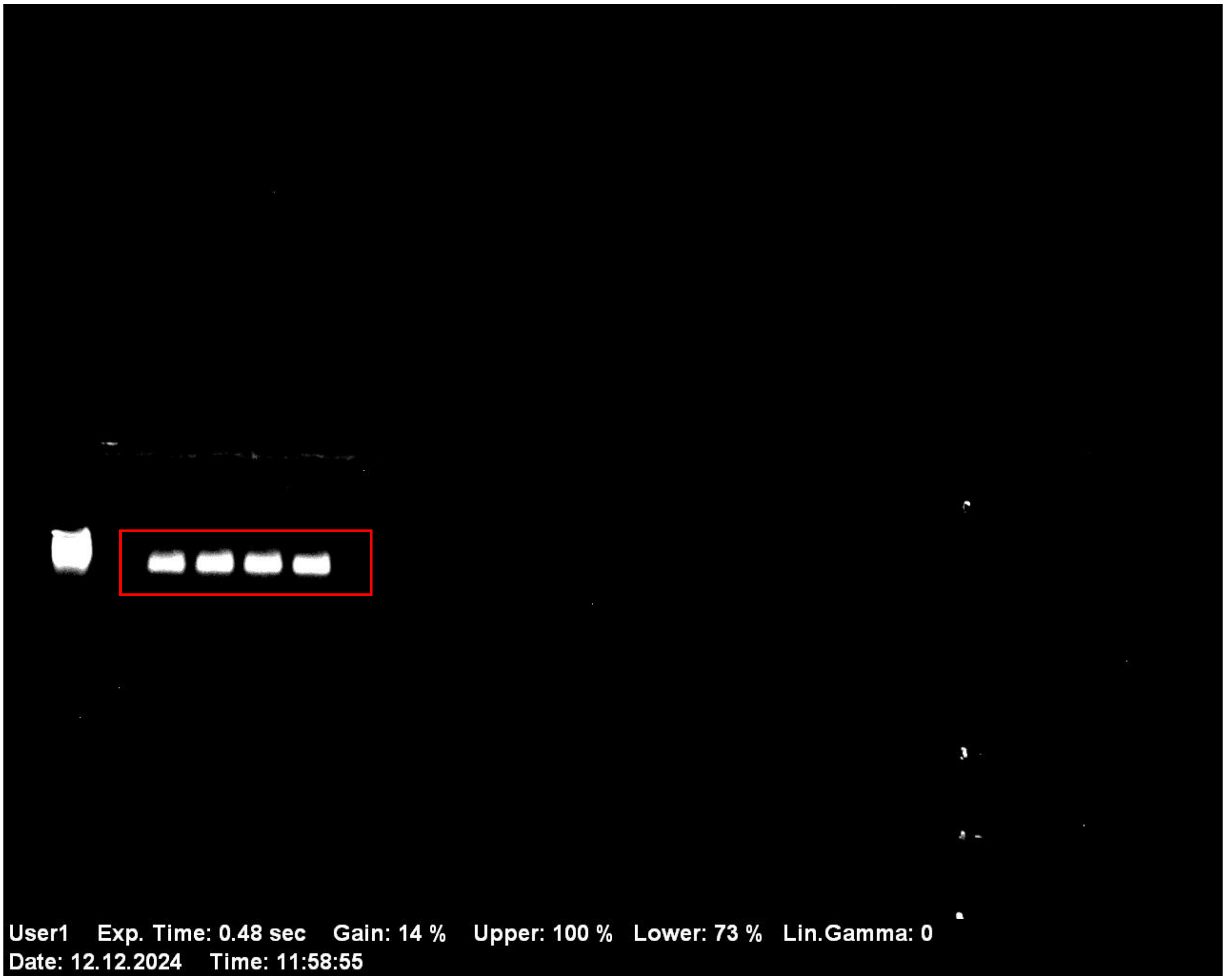

KO

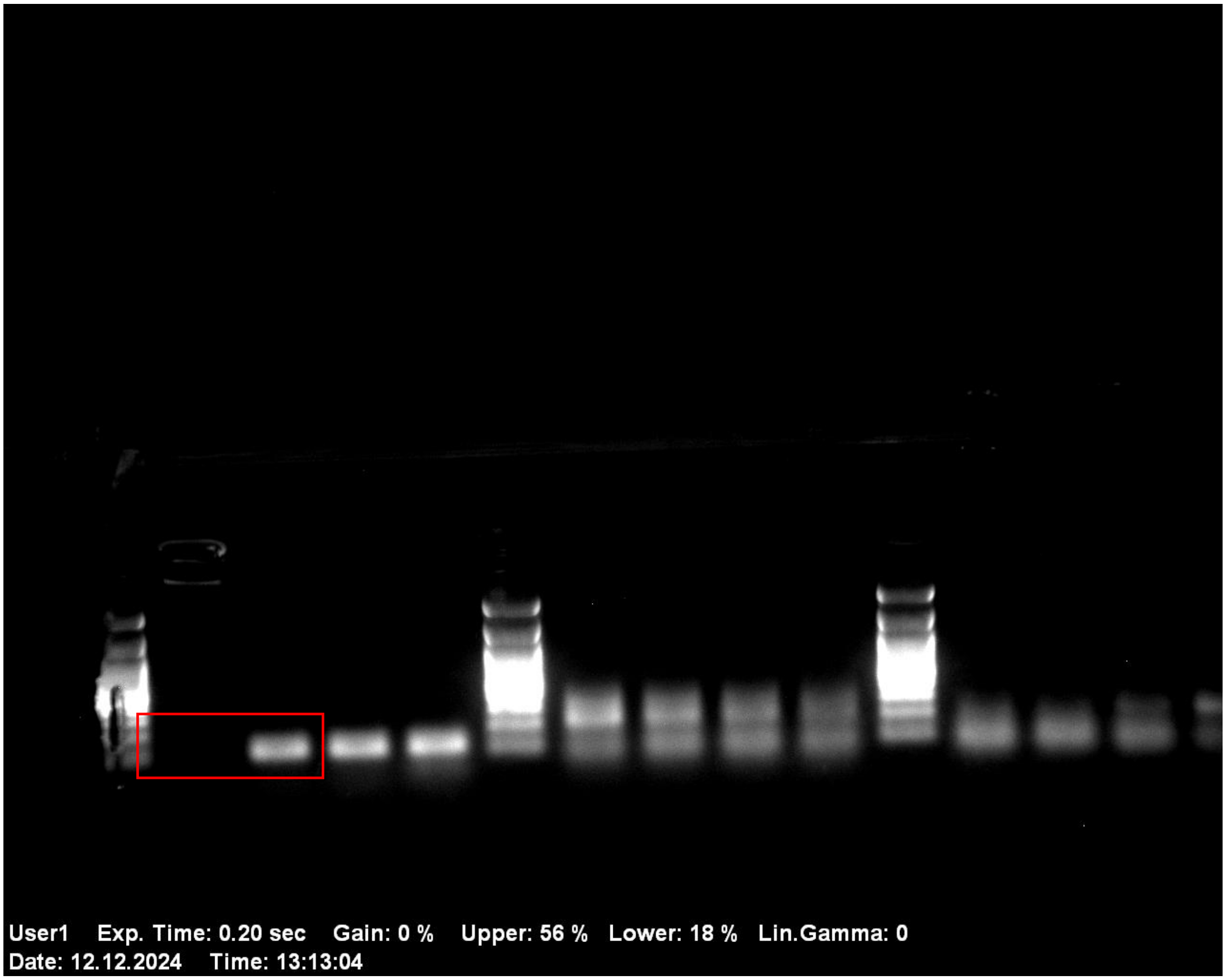
User1 Exp. Time: 0.20 sec Gain: 0 % Upper: 56 % Lower: 18 % Lin.Gamma: 0
Date: 12.12.2024 Time: 13:13:04

OE

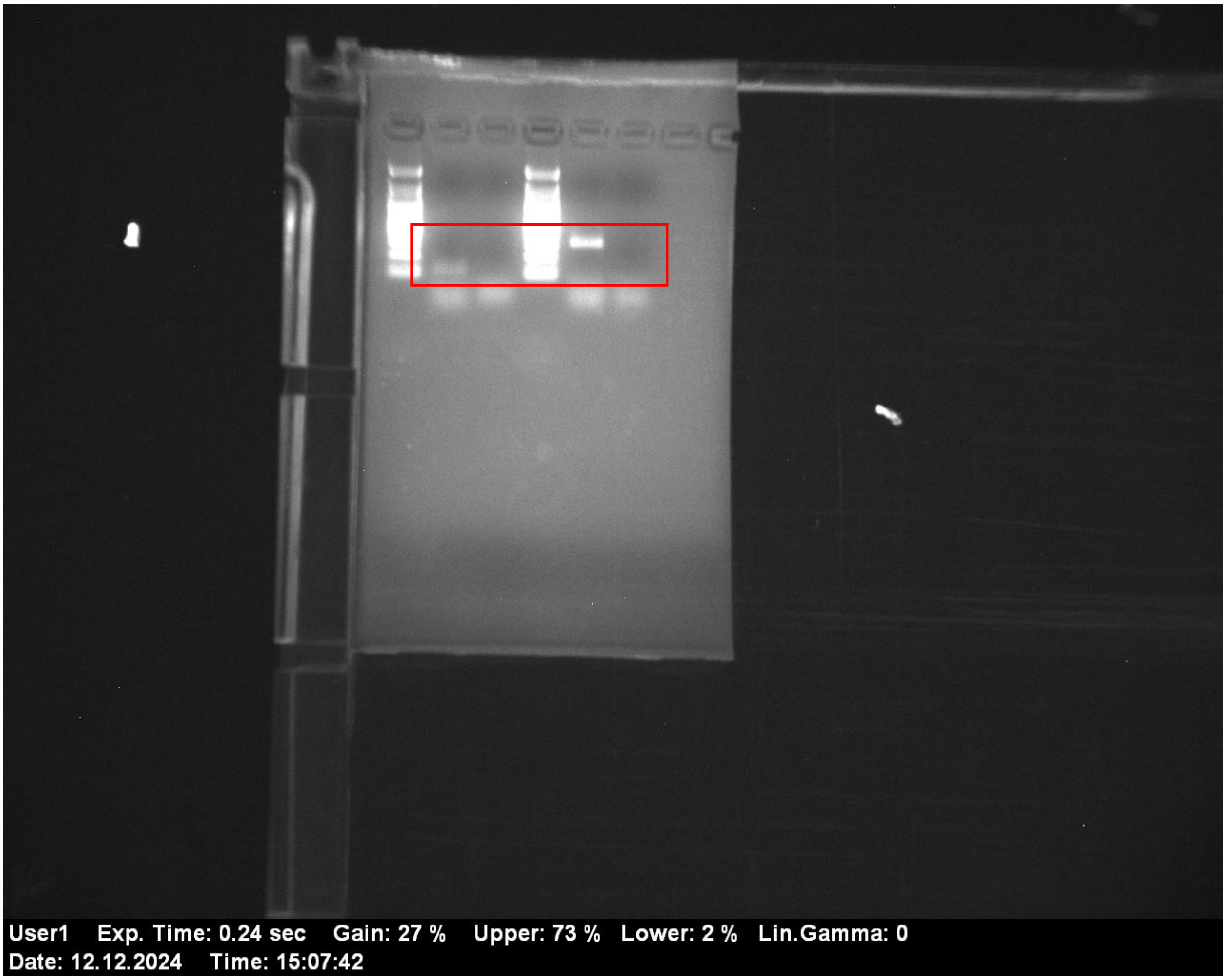
User1 Exp. Time: 0.24 sec Gain: 27 % Upper: 73 % Lower: 2 % Lin.Gamma: 0
Date: 12.12.2024 Time: 15:07:42

Reagents and tools table

Reagent type (species) or resource	Source	Identifiers	Additional information
Rabbit monoclonal anti-ß-Actin (13E5)	Cell signaling	4970	RRID:AB_2223172 1:1000
Mouse monoclonal anti-FTL	novusbio	NBP2-37243	1:500
Rabbit polyclonal anti-FABP5	Cusabio	CSB-PA007946ESR1HU	1:500
Rabbit polyclonal anti-FABP5	Thermo Fisher Scientific	12348-1-AP	RRID:AB_2100341 1:2000
Rabbit polyclonal anti-FABP5	Thermo Fisher Scientific	PA5-79232	1:500
Rabbit polyclonal anti- PrP^C	Sigma	HPA043398	1:100; 1:1000
Mouse monoclonal anti- PrP^C	r-biopharm ag	R8005	1:300
Rabbit monoclonal anti-RAC3	abcam	ab129062	1:1000; 1:300
Rabbit polyclonal anti-GPX8	Proteintech	16846-1-AP	1:1000
Rabbit polyclonal anti-GPX8	GeneTex	GTX125992	1:1000
Rabbit polyclonal anti-FTH1	Cusabio	CSB-PA008485	1:500
Mouse monoclonal anti-HMOX1	Proteintech	66743-1-Ig	1:1000
Rabbit polyclonal anti-COX2	invitrogen	PA5-122071	1:1000
Mouse monoclonal anti-4-hydroxynonenal	R&D systems	MAB3249	1:1000
Rabbit polyclonal anti-TFRC	Atlas	HPA028598	1:1000
Rabbit monoclonal anti-Synapsin	Cell signaling	P17600	1:500
Mouse monoclonal anti-MAP2	Elabscience	E-AB-22030	1:100
Goat anti rabbit IgG Cy3	Jackson Immuno	111-165-003	RRID:AB_2338000 1:500
Goat anti rabbit IgG Cy2	Jackson Immuno	111-225-003	1:200
Anti-rabbit IgG, HRP-linked Antibody	Cell signaling	7074	1:2000
Anti-mouse IgG, HRP-linked Antibody	Cell signaling	7076	1:2000
Rabbit polyclonal anti 4-HNE	Abcam	ab46545	1:1000
XL1-Blue Competent Cells	Agilent	Cat# 200228	
One Shot Stbl3 Chemically Competent E. coli	Thermo Fisher Scientific	Cat# C737303	
Alpha-tocopherol	Sigma	T3251-5G	
Imidazole ketone erastin (IKE)	Stockwell lab		
(1S,3R)-RSL3	Stockwell lab		
BODIPY 581/591 C11	Thermo Fisher Scientific	Cat# D3861	
2,7-Dichlorodihydrofluorescein diacetate (DCFH-DA)	Biomol	Cay85155-50	
Deferoxamine	Sigma	D9533-1G	
Ferric ammonium citrate	Sigma	F5879-100G	

Calcein-AM	Santa Cruz	sc-203865	
FerroOrange Live cellDye	Sigma	SCT210-35NMOL	
Ferrostatin-1	Sigma	SML0583-5mg	
Formaldehyde solution	Sigma	Cat# 47608	
zVAD	Santa Cruz	sc-3067	
Necrostatin-1	abcam	ab141053-5mg	
EHOP-016	Sigma	SML0526-5mg	
DTT	Roth	6908.2	
Staurosporine	Biomol	Cay81590-250	
Etoposide	J&K	Cat# 320523	
CIS-PLATINUM(II)DIAMMINE DICHLORIDE	Sigma	P4394-25MG	
Cyclophosphamide monohydrate (CP)	Th. Geyer	11800456	
Cytochalasin E (CC)	Sigma	Cat# C2149	
Cycloheximide (CHX)	Merck	Cat# 239764	
6-Thioguanine (6-TG)	Sigma	Cat# A4882	
Human: HEK 293T, fetal	ATCC	Cat# CRL-3216	RRID: CVCL_0063
Human: HT-1080, male	ATCC	Cat# CCL-121	RRID: CVCL_0317
Mouse: HT-22		SCC129	
psPAX2	Addgene	Cat# 12259	RRID:Addgene_12260
pMD2g	Addgene	Cat# 12260	RRID:Addgene_12260
lentiCRISPRv2	Addgene	Cat# 52961	RRID:Addgene_52961
pLV hU6-sgRNA hUbC-dCas9-KRAB-T2a-Puro	Addgene	Cat# 71236	
pLV hU6 sgRNA hUbC dCas9 KRAB T2a Neo	This paper		
pLVTHM IRES Puro	This paper		
pLVTHM hPRNP IRES Puro	This paper		
pLVTHM hGPX8 IRES Puro	This paper		
pLV C1-Hyper3 ER	This paper		
pLV hU6_hRAC3_sgRNA hUbC dCas9 KRAB T2a Neo	This paper		
DMEM, high glucose, pyruvate, no glutamine	Thermo Fisher Scientific	Cat# 21969035	
Fetal Bovine Serum	Thermo Fisher Scientific	Cat# 10270106	
MEM Non-essential Amino Acid Solution (100×)	Sigma	Cat# M7145	
L-Glutamine (200 mM)	Thermo Fisher Scientific	Cat# 25030024	
Penicillin-Streptomycin (10,000 U/mL)	Thermo Fisher Scientific	Cat# 15140122	
Puromycin dihydrochloride	Sigma	Cat# P9620; CAS: 58-58-2	

Geneticin Selective Antibiotic (G418 Sulfate)	LifeTechnologies	10131027	
X-tremeGENE HP DNA Transfection Reagent	Sigma	Cat# 6366244001	
Accutase solution	Sigma	Cat# A6964	
BCA kit	Thermo Fisher Scientific	Cat# 22663, Cat#22660	
Skim milk powder	Sigma	Cat# 70166-500G	
PVDF-Membran	Roth	T830.1	
ROTIPHORESE®Gel 30 (37.5:1)	Roth	3029.1	
Ammonium persulfate	Sigma	248614-100G	
TEMED	Roth	2367.3	
Rotiphorese® 10x SDS-PAGE	11653013	Th Geyer	
Sodium Dodecyl Sulphate	AppliChem	A0676,0250	
ECL	Bio-RAD	Cat# 1705060	
AMV Reverse Transcriptase Kit	NEB	M0277S	
Power SYBR Green Master Mix	Lager	5000989	
GraphPad Prism	GraphPad Software	www.graphpad.com	
FlowJo 10	FlowJo LLC	www.flowjo.com	
Columbus 2.9.1.532	PerkinElmer		
Harmony software			

CRISPR guide sequences and Primers

**CRISPR guide sequences**	**SOURCE**	**IDENTIFIER**
Human *RAC3* Knockdown guide CACCGCAGCTCTGCCCGGGTTCGGG	This paper	N/A
Human *GPX8* Knockout guide 1 CACCGTGGGCCCGGAACATTTTAGC	This paper	N/A
Human *GPX8* Knockout guide 2 CACCGGTCTGTGAGTTGGCAGTCAC	This paper	N/A
Human *GPX8* Knockdown guide 1 CACCGCGAAACTCCTGAATGAAGCA	This paper	N/A
Human *GPX8* Knockdown guide 2 CACCGAGCAGTCAGCCTGTCCTTCC	This paper	N/A
Human *GPX8* Knockdown guide 3 CACCGACAGAAAGACCACTTCTCAG	This paper	N/A
Mouse *Prnp* Knockout guide 1 CACCGCCTGGAGGGTGGAACACCGG	This paper	N/A
Mouse *Prnp* Knockout guide 2 CACCGACCTGGGGGCAGCCCCACGG	This paper	N/A
**Cloning primers**		
Human *PRNP* amplification forward primer: CGAGACTAGCCTCGAGGTTTAAACGCCACCATGGCGAACC TTGGCTGCTGGATG	This paper	N/A

Human *PRNP* amplification reverse primer: GCCTTCACAAAGATCCTCATATGTCATCCCACTATCAGGAAGATGAGG	This paper	N/A
Human *GPX8* amplification forward primer: CGAGACTAGCCTCGAGGTTTAAACGCCACCATGGAGCCTCTTGCAGCTTACC	This paper	N/A
Human *GPX8* amplification reverse primer: GCCTTCACAAAGATCCTCATATGTCATAGATCCTCTTTCTTTTTTATGATC	This paper	N/A
Human *RAC3* amplification forward 1 primer: CGAGACTAGCCTCGAGGTTTAAACGCCACCATGCAGGCCATCAAGTGCGTGG	This paper	N/A
Human *RAC3* amplification reverse 1 primer: CACAAAGATCCTCATATGCTAGAAGACGGTGCACTTCTTC	This paper	N/A
Human *RAC3* amplification forward 2 primer: AGCATATGAGGATCTTTGTGAAGGCAATTCCGCCCCCCCC	This paper	N/A
Human *RAC3* amplification reverse 2 primer: TGTAATCCAGAGGTTGATTATTCAGGCACCGGGCTTGCGGGT	This paper	N/A
**qPCR primers**		
Human *PRNP* qPCR forward primer: AGTCAGTGGAACAAGCCGAG	This paper	N/A
Human *PRNP* qPCR reverse primer: TGGCACTTCCCAGCATGTAG	This paper	N/A
Human *RAC3* qPCR forward primer: ACAAGGACACCATTGAGCGGCT	This paper	N/A
Human *RAC3* qPCR reverse primer: CCTCGTCAAACACTGTCTTCAGG	This paper	N/A
Human *GPX8* qPCR forward primer: CTACGGAGTAACTTTCCCCATCTTCCACAAG	This paper	N/A
Human *GPX8* qPCR reverse primer: CTGCTATGTCAGGCCTGATGACTTCAATGG	This paper	N/A
Human *TEAD1* qPCR forward primer: CCTGGCTATCTATCCACCATGTG	This paper	N/A
Human *TEAD1* qPCR reverse primer: TTCTGGTCCTCGTCTTGCCTGT	This paper	N/A
Human *ZEB1* qPCR forward primer: GGCATACACCTACTCAACTACGG	This paper	N/A
Human *ZEB1* qPCR reverse primer:	This paper	N/A

TGGGCGGTGTAGAATCAGAGTC		
Human *SLUG* qPCR forward primer: ATCTGCGGCAAGGCGTTTTCCA	This paper	N/A
Human *SLUG* qPCR reverse primer: GAGCCCTCAGATTTGACCTGTC	This paper	N/A
Human *NCAD* qPCR forward primer: CCTCCAGAGTTTACTGCCATGAC	This paper	N/A
Human *NCAD* qPCR reverse primer: GTAGGATCTCCGCCACTGATTC	This paper	N/A
Human *VIMENTIN* qPCR forward primer: AGGCAAAGCAGGAGTCCACTGA	This paper	N/A
Human *VIMENTIN* qPCR reverse primer: ATCTGGCGTTCCAGGGACTCAT	This paper	N/A
Human *ECAD* qPCR forward primer: CAATGCCGCCATCGCTTAC	This paper	N/A
Human *ECAD* qPCR reverse primer: ATGACTCCTGTGTTCCTGTTAATG	This paper	N/A
Human *SNAIL* qPCR forward primer: TTTCTGGTTCTGTGTCCTCTGCCT	This paper	N/A
Human *SNAIL* qPCR reverse primer: TGAGTCTGTCAGCCTTTGTCCTGT	This paper	N/A
Human *TBP* qPCR forward primer: GCGGTTTGCTGCGGTAATC	This paper	N/A
Human *TBP* qPCR reverse primer: CTTCACTCTTGGCTCCTGTGC	This paper	N/A
Human *RPL27* qPCR forward primer: TCGCCAAGAGATCAAAGATAA	This paper	N/A
Human *RPL27* qPCR reverse primer: CTGAAGACATCCTTATTGACG	This paper	N/A
Human *FABP5* qPCR forward primer: GCAGACCCCTCTCTGCAC	This paper	N/A

Human *FABP5* qPCR reverse primer: TCGCAAAGCTATTCCCACTC	This paper	N/A
Human *PTGS2* qPCR forward primer: TGGAGCACCATTCTCCTTGAAAGGACTTAT	This paper	N/A
Human *PTGS2* qPCR reverse primer: GACTGTTTTAATGAGCTCTGGATCTGGAAC	This paper	N/A
Human *TFRC* qPCR forward primer: ACACGCTGCCAGCTTTACTGGAGAACTT	This paper	N/A
Human *TFRC* qPCR reverse primer: AGAGGGCATTTGCAGCTCCCTGAATA	This paper	N/A
Human *HMOX1* qPCR forward primer: TTCTTCACCTTCCCCAACATT	This paper	N/A
Human *HOMX1* qPCR reverse primer: CAGCTCCTGCAACTCCTCAAA	This paper	N/A
Human *GPX4* qPCR forward primer: GCCTTCCCGTGTAACCAGT	This paper	N/A
Human *GPX4* qPCR reverse primer: GCGAACTCTTTGATCTCTTCGT	This paper	N/A
Mouse *bActin* qPCR forward primer: CCTCTATGCCAACACAGTGC	This paper	N/A
Mouse *bActin* qPCR reverse primer: GTACTCCTGCTTGCTGATCC	This paper	N/A

Reagents and tools table. Reagents and tools used in this paper.
