# Supplementary material for: Prion-induced ferroptosis is facilitated by RAC3
Source: Nat Commun. 2025 Jun 25;16:5385. doi: 10.1038/s41467-025-60793-3 (PMC12198409; doi:10.1038/s41467-025-60793-3)
Supplement: Supplementary file 4 — Source data [file 41467_2025_60793_MOESM4_ESM.zip › Reagents and Tools Table.pdf]

Reagent type (species) or resource	Source	Identifiers	Additional information
Rabbit monoclonal anti-ß-Actin (13E5)	Cell signaling	4970	RRID:AB_2223172 1:1000
Mouse monoclonal anti-FTL	novusbio	NBP2-37243	1:500
Rabbit polyclonal anti-FABP5	Cusabio	CSB-PA007946ESR1HU	1:500
Rabbit polyclonal anti-FABP5	Thermo Fisher Scientific	12348-1-AP	RRID:AB_2100341 1:2000
Rabbit polyclonal anti-FABP5	Thermo Fisher Scientific	PA5-79232	1:500
Rabbit polyclonal anti- PrP^C	Sigma	HPA043398	1:100; 1:1000
Mouse monoclonal anti- PrP^C	r-biopharm ag	R8005	1:300
Rabbit monoclonal anti-RAC3	abcam	ab129062	1:1000; 1:300
Rabbit polyclonal anti-GPX8	Proteintech	16846-1-AP	1:1000
Rabbit polyclonal anti-GPX8	GeneTex	GTX125992	1:1000
Rabbit polyclonal anti-FTH1	Cusabio	CSB-PA008485	1:500
Mouse monoclonal anti-HMOX1	Proteintech	66743-1-Ig	1:1000
Rabbit polyclonal anti-COX2	invitrogen	PA5-122071	1:1000
Mouse monoclonal anti-4-hydroxynonenal	R&D systems	MAB3249	1:1000
Rabbit polyclonal anti-TFRC	Atlas	HPA028598	1:1000
Rabbit monoclonal anti-Synapsin	Cell signaling	P17600	1:500
Mouse monoclonal anti-MAP2	Elabscience	E-AB-22030	1:100
Goat anti rabbit IgG Cy3	Jackson Immuno	111-165-003	RRID:AB_2338000 1:500
Goat anti rabbit IgG Cy2	Jackson Immuno	111-225-003	1:200
Anti-rabbit IgG, HRP-linked Antibody	Cell signaling	7074	1:2000
Anti-mouse IgG, HRP-linked Antibody	Cell signaling	7076	1:2000
Rabbit polyclonal anti 4-HNE	Abcam	ab46545	1:1000
XL1-Blue Competent Cells	Agilent	Cat# 200228	
One Shot Stbl3 Chemically Competent E. coli	Thermo Fisher Scientific	Cat# C737303	
Alpha-tocopherol	Sigma	T3251-5G	
Imidazole ketone erastin (IKE)	Stockwell lab		
(1S,3R)-RSL3	Stockwell lab		
BODIPY 581/591 C11	Thermo Fisher Scientific	Cat# D3861	
2,7-Dichlorodihydrofluorescein diacetate (DCFH-DA)	Biomol	Cay85155-50	
Deferoxamine	Sigma	D9533-1G	
Ferric ammonium citrate	Sigma	F5879-100G	
Calcein-AM	Santa Cruz	sc-203865	
FerroOrange Live cellDye	Sigma	SCT210-35NMOL	
Ferrostatin-1	Sigma	SML0583-5mg	

Formaldehyde solution	Sigma	Cat# 47608	
zVAD	Santa Cruz	sc-3067	
Necrostatin-1	abcam	ab141053-5mg	
EHOP-016	Sigma	SML0526-5mg	
DTT	Roth	6908.2	
Staurosporine	Biomol	Cay81590-250	
Etoposide	J&K	Cat# 320523	
CIS-PLATINUM(II)DIAMMINE DICHLORIDE	Sigma	P4394-25MG	
Cyclophosphamide monohydrate (CP)	Th. Geyer	11800456	
Cytochalasin E (CC)	Sigma	Cat# C2149	
Cycloheximide (CHX)	Merck	Cat# 239764	
6-Thioguanine (6-TG)	Sigma	Cat# A4882	
Human: HEK 293T, fetal	ATCC	Cat# CRL-3216	RRID: CVCL_0063
Human: HT-1080, male	ATCC	Cat# CCL-121	RRID: CVCL_0317
Mouse: HT-22		SCC129	
psPAX2	Addgene	Cat# 12259	RRID:Addgene_12260
pMD2g	Addgene	Cat# 12260	RRID:Addgene_12260
lentiCRISPRv2	Addgene	Cat# 52961	RRID:Addgene_52961
pLV hU6-sgRNA hUbC-dCas9-KRAB-T2a-Puro	Addgene	Cat# 71236	
pLV hU6 sgRNA hUbC dCas9 KRAB T2a Neo	This paper		
pLVTHM IRES Puro	This paper		
pLVTHM hPRNP IRES Puro	This paper		
pLVTHM hGPX8 IRES Puro	This paper		
pLV C1-Hyper3 ER	This paper		
pLV hU6_hRAC3_sgRNA hUbC dCas9 KRAB T2a Neo	This paper		
DMEM, high glucose, pyruvate, no glutamine	Thermo Fisher Scientific	Cat# 21969035	
Fetal Bovine Serum	Thermo Fisher Scientific	Cat# 10270106	
MEM Non-essential Amino Acid Solution (100×)	Sigma	Cat# M7145	
L-Glutamine (200 mM)	Thermo Fisher Scientific	Cat# 25030024	
Penicillin-Streptomycin (10,000 U/mL)	Thermo Fisher Scientific	Cat# 15140122	
Puromycin dihydrochloride	Sigma	Cat# P9620; CAS: 58-58-2	
Geneticin Selective Antibiotic (G418 Sulfate)	LifeTechnologies	10131027	
X-tremeGENE HP DNA Transfection Reagent	Sigma	Cat# 6366244001	

Accutase solution	Sigma	Cat# A6964	
BCA kit	Thermo Fisher Scientific	Cat# 22663, Cat#22660	
Skim milk powder	Sigma	Cat# 70166-500G	
PVDF-Membran	Roth	T830.1	
ROTIPHORESE®Gel 30 (37.5:1)	Roth	3029.1	
Ammonium persulfate	Sigma	248614-100G	
TEMED	Roth	2367.3	
Rotiphorese® 10x SDS-PAGE	11653013	Th Geyer	
Sodium Dodecyl Sulphate	AppliChem	A0676,0250	
ECL	Bio-RAD	Cat# 1705060	
AMV Reverse Transcriptase Kit	NEB	M0277S	
Power SYBR Green Master Mix	Lager	5000989	
GraphPad Prism	GraphPad Software	www.graphpad.com	
FlowJo 10	FlowJo LLC	www.flowjo.com	
Columbus 2.9.1.532	PerkinElmer		
Harmony software			

CRISPR guide sequences and Primers

CRISPR guide sequences	SOURCE	IDENTIFIER
Human *RAC3* Knockdown guide CACCGCAGCTCTGCCCGGGTTCGGG	This paper	N/A
Human *GPX8* Knockout guide 1 CACCGTGGGCCCGGAACATTTTAGC	This paper	N/A
Human *GPX8* Knockout guide 2 CACCGGTCTGTGAGTTGGCAGTCAC	This paper	N/A
Human *GPX8* Knockdown guide 1 CACCGCGAAACTCCTGAATGAAGCA	This paper	N/A
Human *GPX8* Knockdown guide 2 CACCGAGCAGTCAGCCTGTCCTTCC	This paper	N/A
Human *GPX8* Knockdown guide 3 CACCGACAGAAAGACCACTTCTCAG	This paper	N/A
Mouse *Prnp* Knockout guide 1 CACCGCCTGGAGGGTGGAACACCGG	This paper	N/A
Mouse *Prnp* Knockout guide 2 CACCGACCTGGGGGCAGCCCCACGG	This paper	N/A
**Cloning primers**		
Human *PRNP* amplification forward primer: CGAGACTAGCCTCGAGGTTTAAACGCCACCATGGCGAACCTTGGCTGCTGGATG	This paper	N/A
Human *PRNP* amplification reverse primer: GCCTTCACAAAGATCCTCATATGTCATCCCACTATCAGGAAGATGAGG	This paper	N/A

Human *GPX8* amplification forward primer: CGAGACTAGCCTCGAGGTTTAAACGCCACCATGGAGCCTCTTGCAGCTTACC	This paper	N/A
Human *GPX8* amplification reverse primer: GCCTTCACAAAGATCCTCATATGTCATAGATCCTCTTTCTTTTTTATGATC	This paper	N/A
Human *RAC3* amplification forward 1 primer: CGAGACTAGCCTCGAGGTTTAAACGCCACCATGCAGGCCATCAAGTGCGTGG	This paper	N/A
Human *RAC3* amplification reverse 1 primer: CACAAAGATCCTCATATGCTAGAAGACGGTGCACTTCTTC	This paper	N/A
Human *RAC3* amplification forward 2 primer: AGCATATGAGGATCTTTGTGAAGGCAATTCCGCCCCCCCC	This paper	N/A
Human *RAC3* amplification reverse 2 primer: TGTAATCCAGAGGTTGATTATTCAGGCACCGGGCTTGCGGGT	This paper	N/A
**qPCR primers**		
Human *PRNP* qPCR forward primer: AGTCAGTGGAACAAGCCGAG	This paper	N/A
Human *PRNP* qPCR reverse primer: TGGCACTTCCCAGCATGTAG	This paper	N/A
Human *RAC3* qPCR forward primer: ACAAGGACACCATTGAGCGGCT	This paper	N/A
Human *RAC3* qPCR reverse primer: CCTCGTCAAACACTGTCTTCAGG	This paper	N/A
Human *GPX8* qPCR forward primer: CTACGGAGTAACTTTCCCCATCTTCCACAAG	This paper	N/A
Human *GPX8* qPCR reverse primer: CTGCTATGTCAGGCCTGATGACTTCAATGG	This paper	N/A
Human *TEAD1* qPCR forward primer: CCTGGCTATCTATCCACCATGTG	This paper	N/A
Human *TEAD1* qPCR reverse primer: TTCTGGTCCTCGTCTTGCCTGT	This paper	N/A
Human *ZEB1* qPCR forward primer: GGCATACACCTACTCAACTACGG	This paper	N/A
Human *ZEB1* qPCR reverse primer: TGGGCGGTGTAGAATCAGAGTC	This paper	N/A
Human *SLUG* qPCR forward primer:	This paper	N/A

ATCTGCGGCAAGGCGTTTTCCA		
Human *SLUG* qPCR reverse primer:<br>GAGCCCTCAGATTTGACCTGTC	This paper	N/A
Human *NCAD* qPCR forward primer:<br>CCTCCAGAGTTTACTGCCATGAC	This paper	N/A
Human *NCAD* qPCR reverse primer:<br>GTAGGATCTCCGCCACTGATTC	This paper	N/A
Human *VIMENTIN* qPCR forward primer:<br>AGGCAAAGCAGGAGTCCACTGA	This paper	N/A
Human *VIMENTIN* qPCR reverse primer:<br>ATCTGGCGTTCCAGGGACTCAT	This paper	N/A
Human *ECAD* qPCR forward primer:<br>CAATGCCGCCATCGCTTAC	This paper	N/A
Human *ECAD* qPCR reverse primer:<br>ATGACTCCTGTGTTCCTGTTAATG	This paper	N/A
Human *SNAIL* qPCR forward primer:<br>TTTCTGGTTCTGTGTCCTCTGCCT	This paper	N/A
Human *SNAIL* qPCR reverse primer:<br>TGAGTCTGTCAGCCTTTGTCCTGT	This paper	N/A
Human *TBP* qPCR forward primer:<br>GCGGTTTGCTGCGGTAATC	This paper	N/A
Human *TBP* qPCR reverse primer:<br>CTTCACTCTTGGCTCCTGTGC	This paper	N/A
Human *RPL27* qPCR forward primer:<br>TCGCCAAGAGATCAAAGATAA	This paper	N/A
Human *RPL27* qPCR reverse primer:<br>CTGAAGACATCCTTATTGACG	This paper	N/A
Human *FABP5* qPCR forward primer:<br>GCAGACCCCTCTCTGCAC	This paper	N/A
Human *FABP5* qPCR reverse primer:<br>TCGCAAAGCTATTCCCACTC	This paper	N/A

Human *PTGS2* qPCR forward primer: TGGAGCACCATTCTCCTTGAAAGGACTTAT	This paper	N/A
Human *PTGS2* qPCR reverse primer: GACTGTTTTAATGAGCTCTGGATCTGGAAC	This paper	N/A
Human *TFRC* qPCR forward primer: ACACGCTGCCAGCTTTACTGGAGAACTT	This paper	N/A
Human *TFRC* qPCR reverse primer: AGAGGGCATTTGCAGCTCCCTGAATA	This paper	N/A
Human *HMOX1* qPCR forward primer: TTCTTCACCTTCCCCAACATT	This paper	N/A
Human *HOMX1* qPCR reverse primer: CAGCTCCTGCAACTCCTCAAA	This paper	N/A
Human *GPX4* qPCR forward primer: GCCTTCCCGTGTAACCAGT	This paper	N/A
Human *GPX4* qPCR reverse primer: GCGAACTCTTTGATCTCTTCGT	This paper	N/A
Mouse *bActin* qPCR forward primer: CCTCTATGCCAACACAGTGC	This paper	N/A
Mouse *bActin* qPCR reverse primer: GTACTCCTGCTTGCTGATCC	This paper	N/A
