# Supplementary material for: Prion-induced ferroptosis is facilitated by RAC3
Source: Nat Commun. 2025 Jun 25;16:5385. doi: 10.1038/s41467-025-60793-3 (PMC12198409; doi:10.1038/s41467-025-60793-3)

Fig 1A

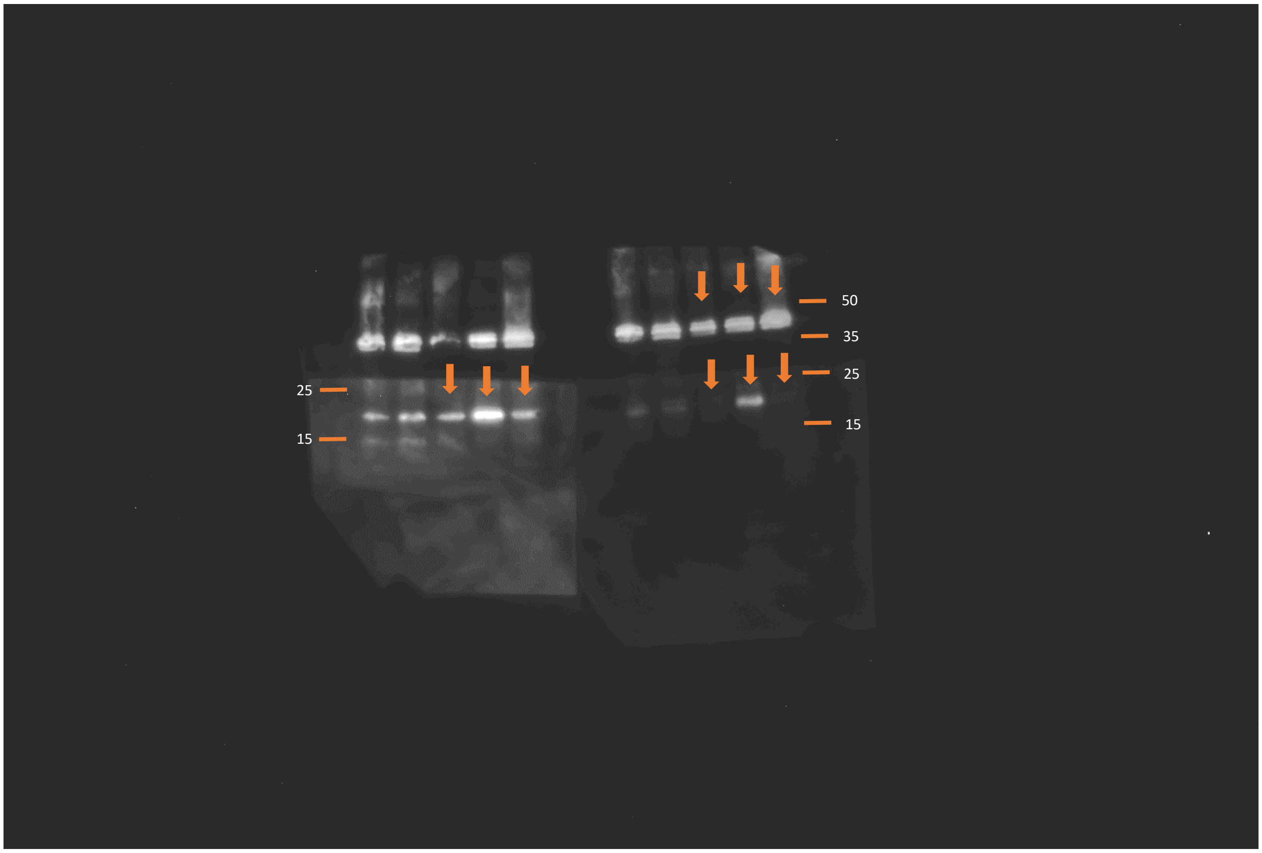

FTH1

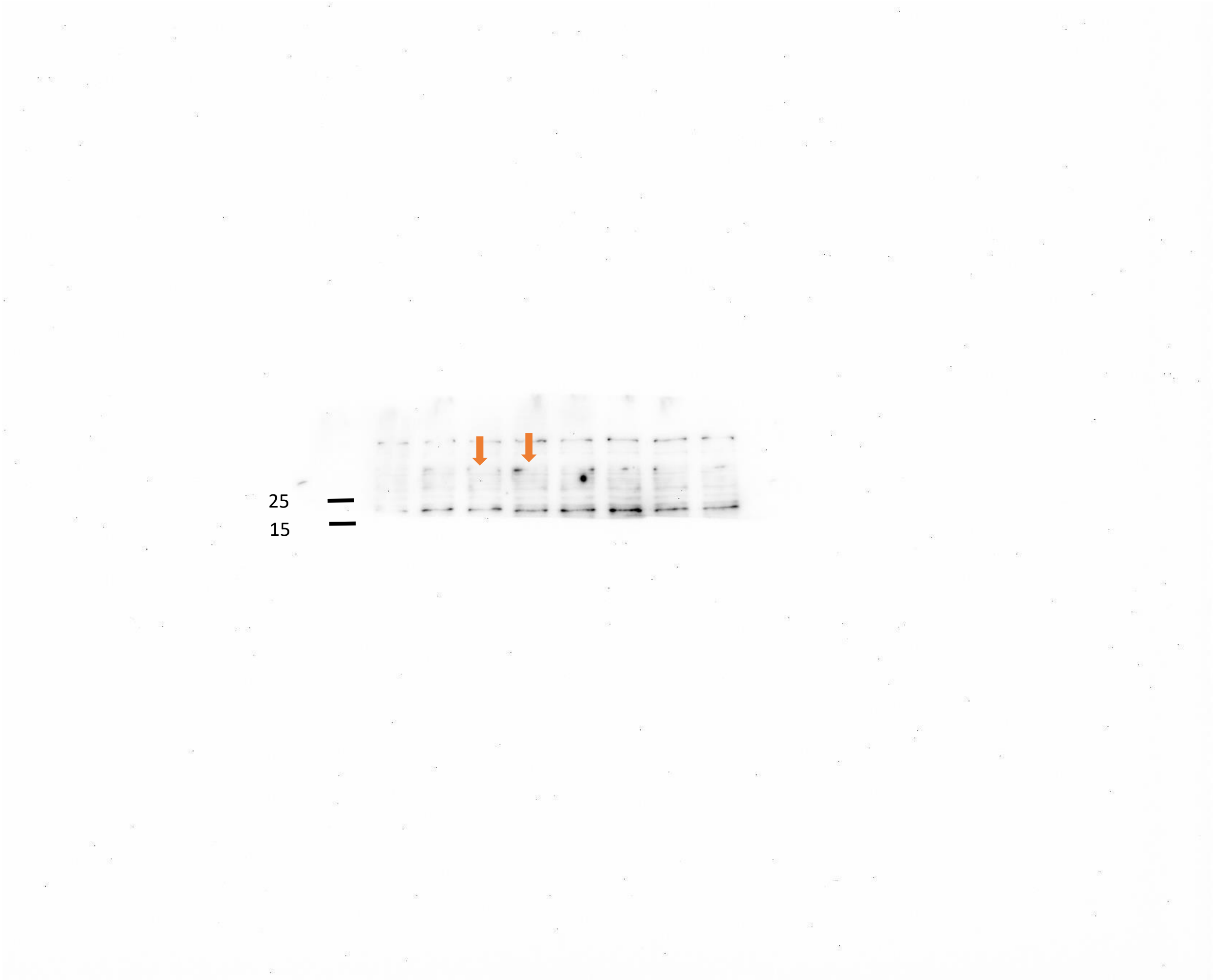

FTL

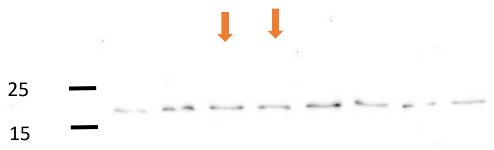

Actin

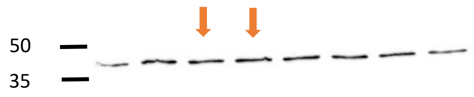

1G Actin

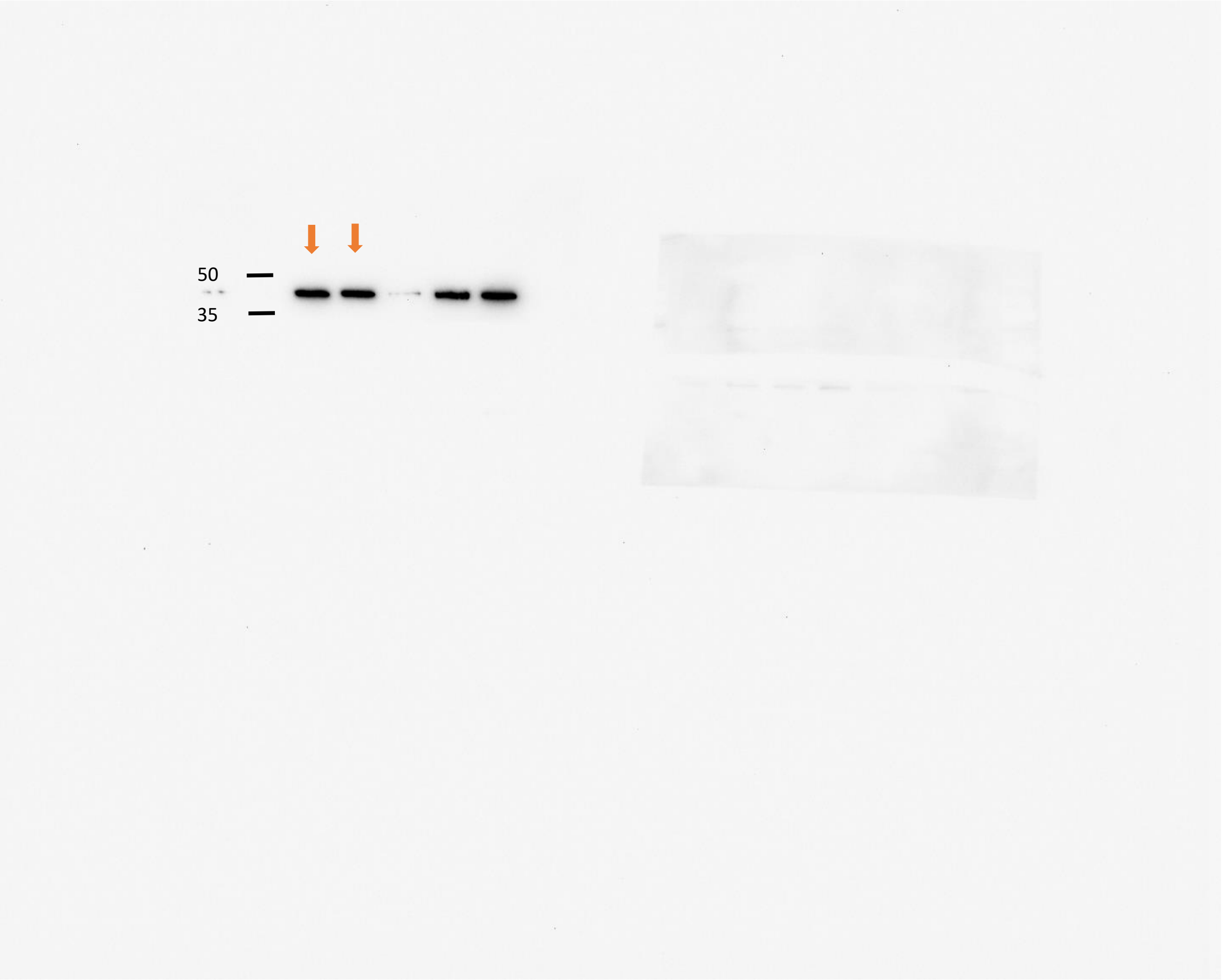

GPX8

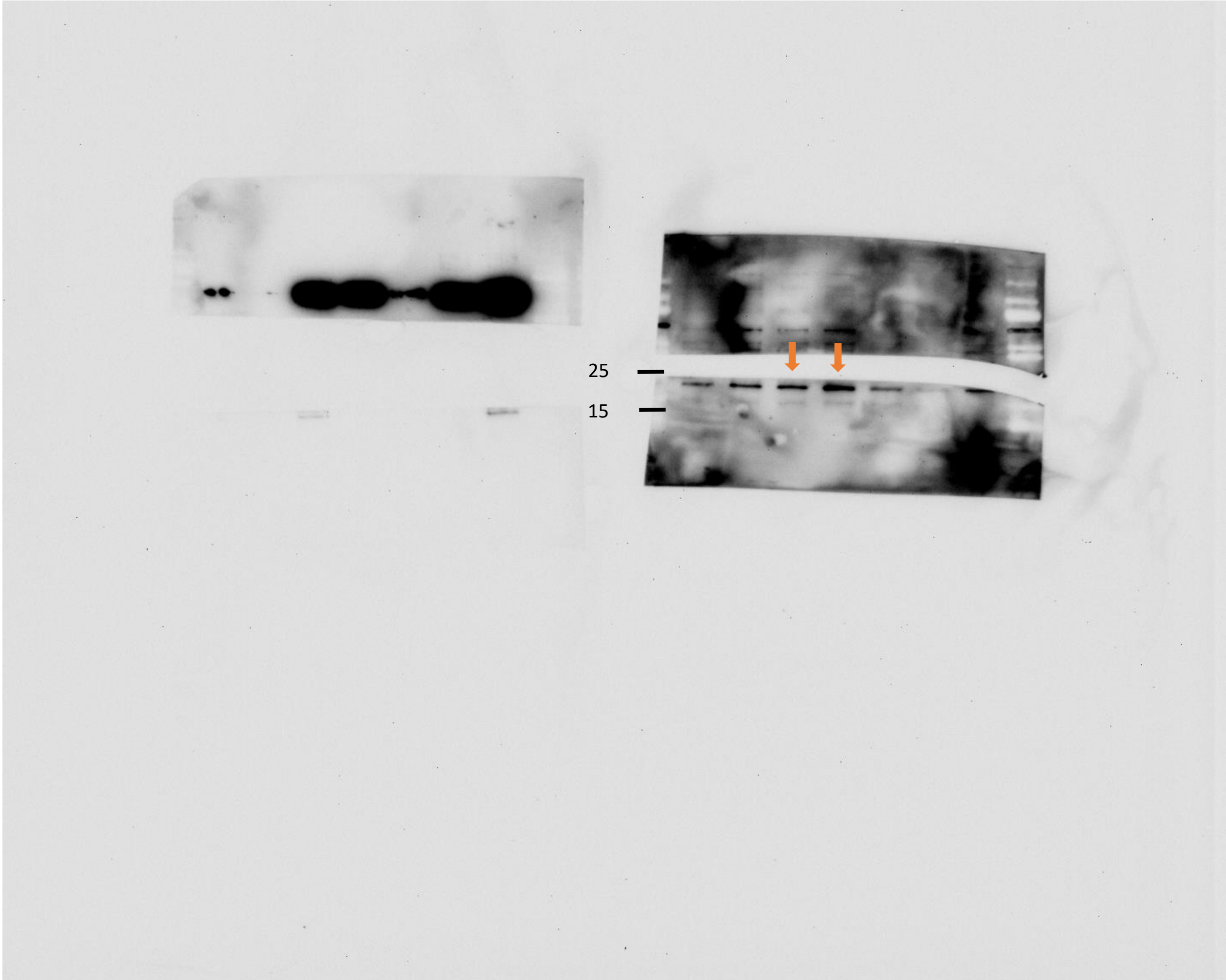

PRNP

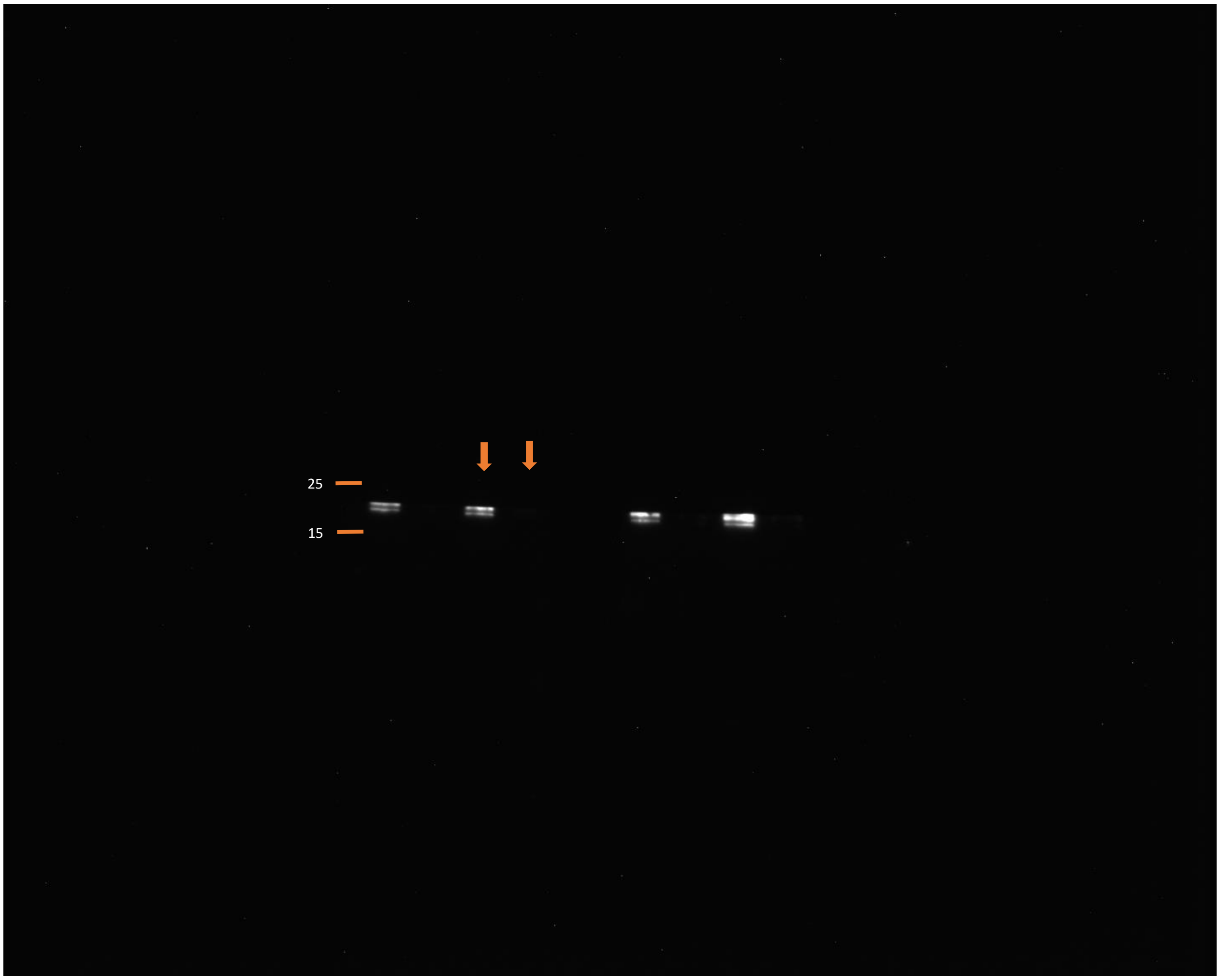

1H Actin

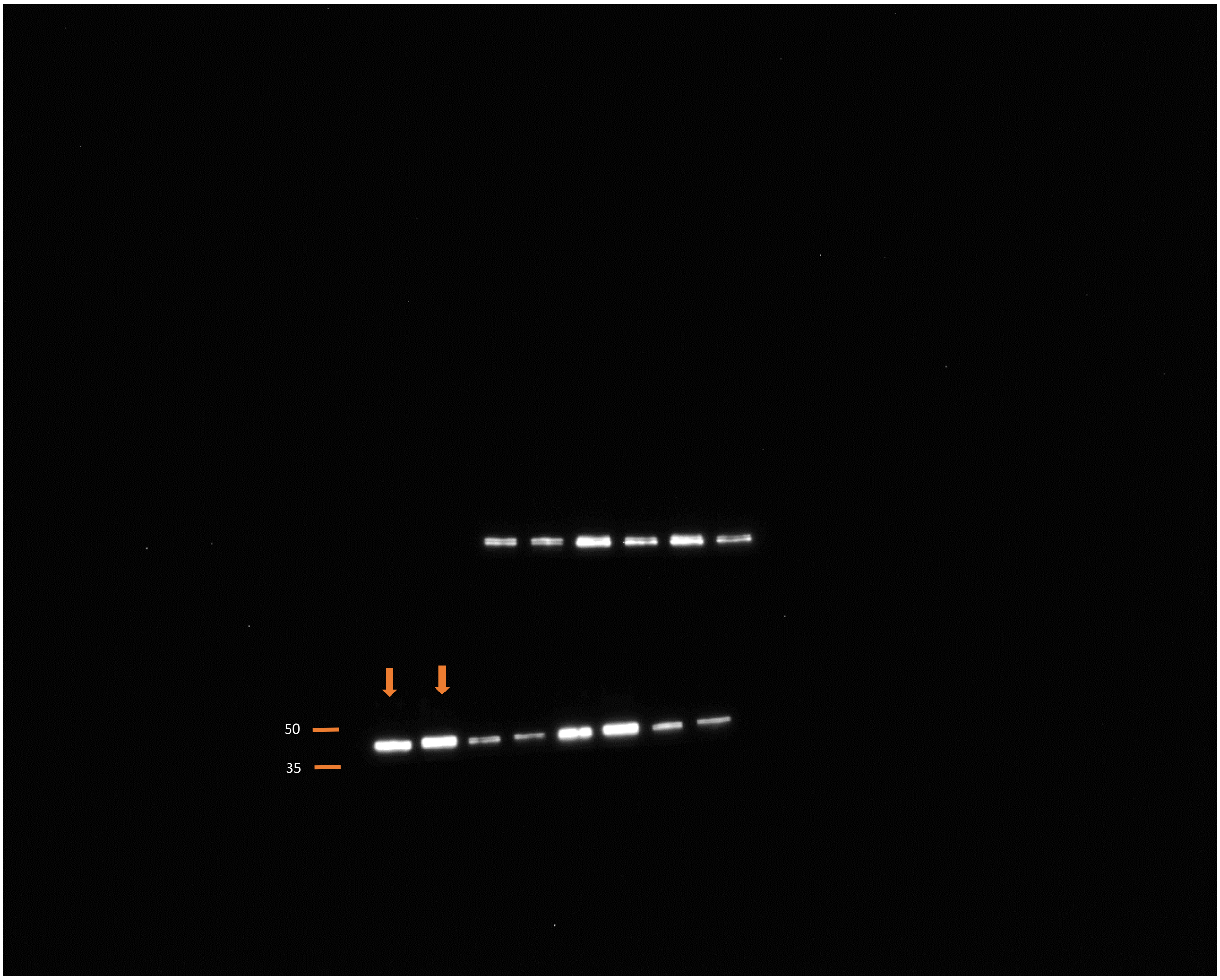

GPX8

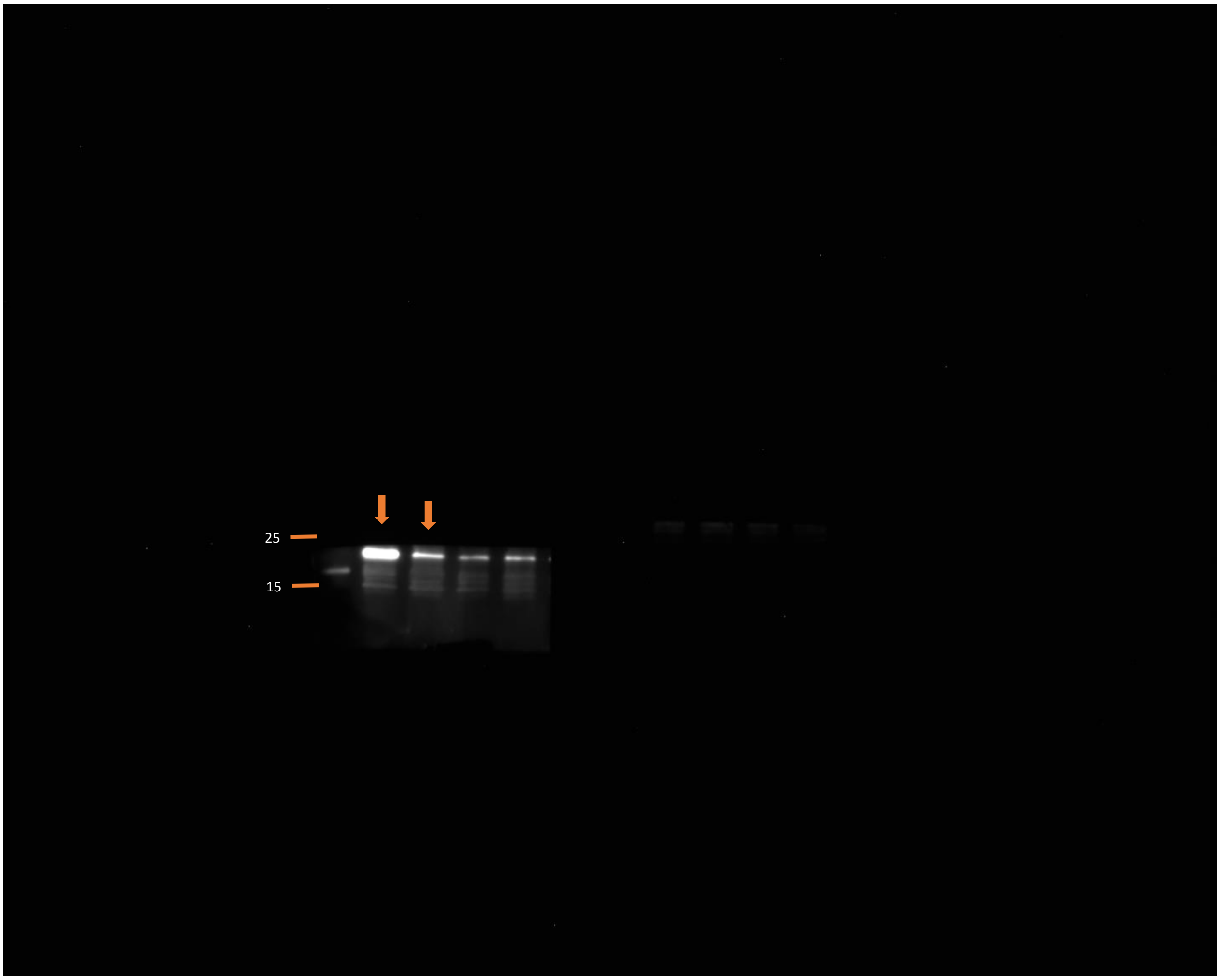

Fig 2B total protein

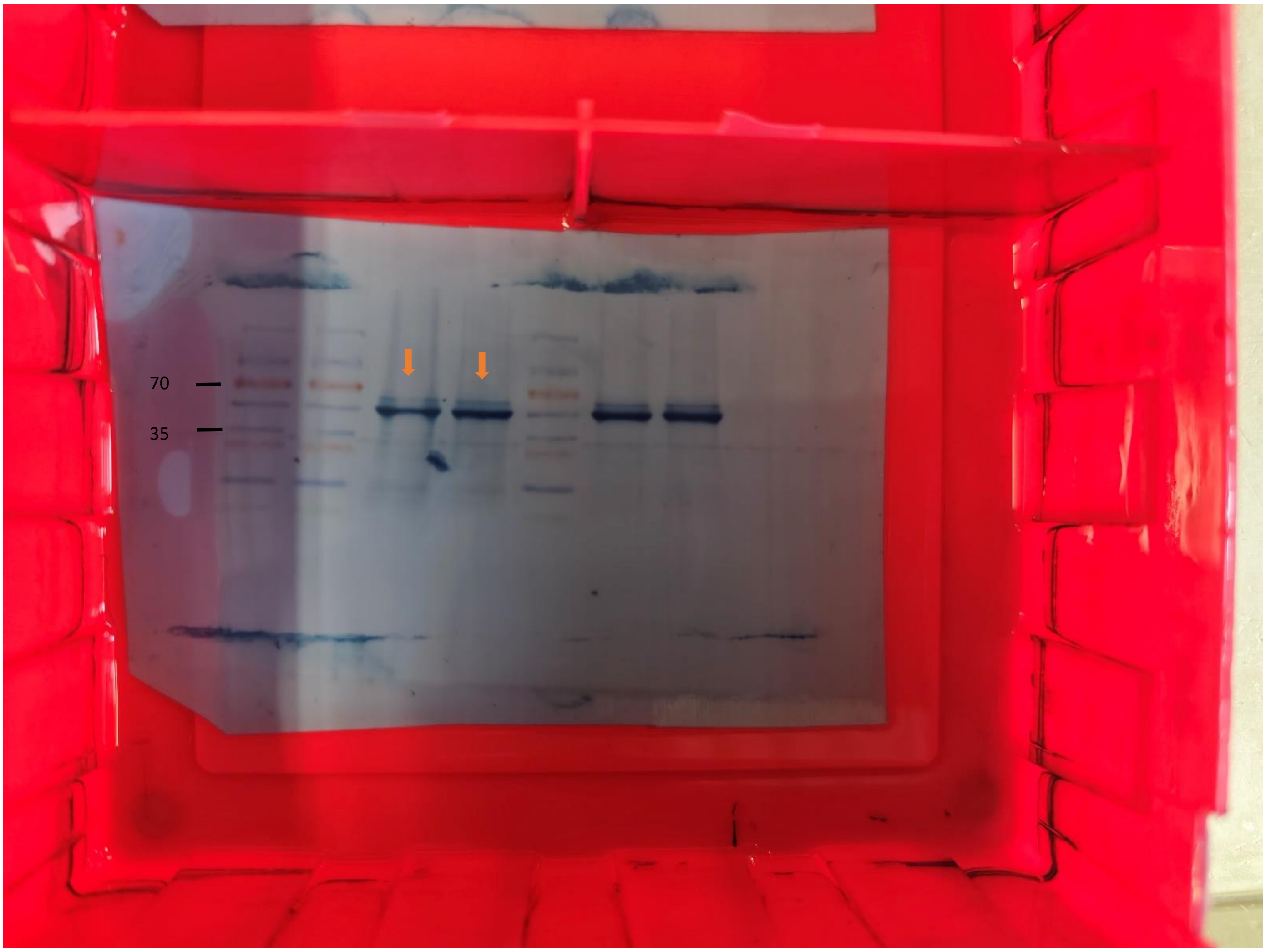

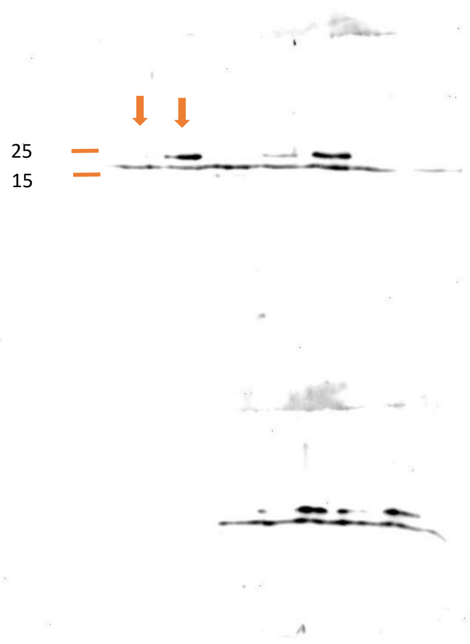
25
15

Fig 3B total protein

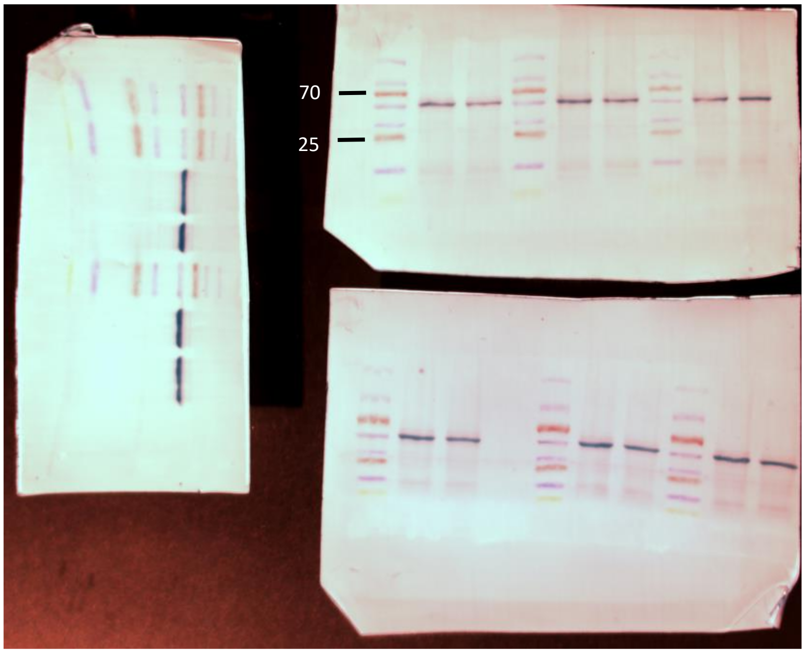

FABP5

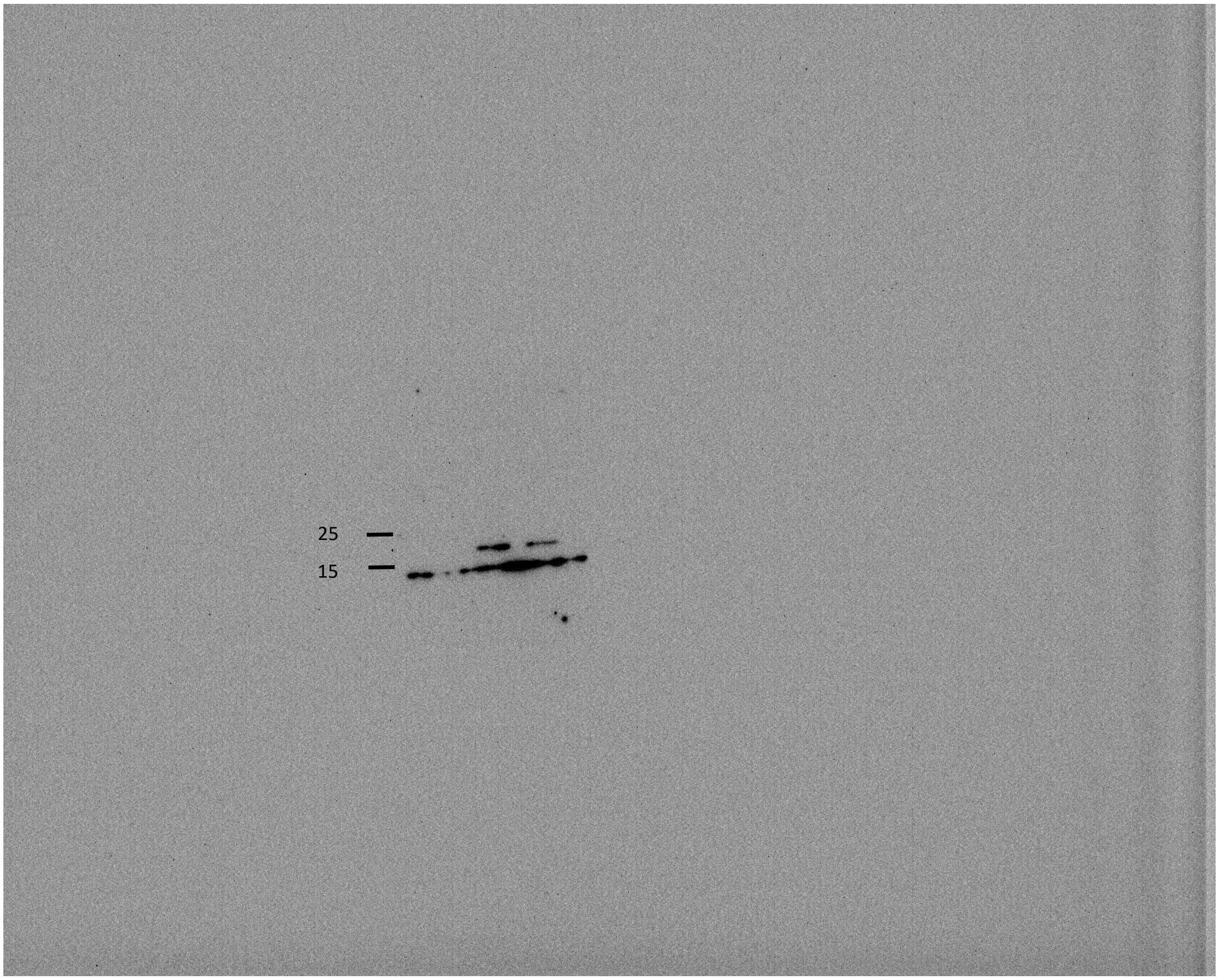

4-HNE

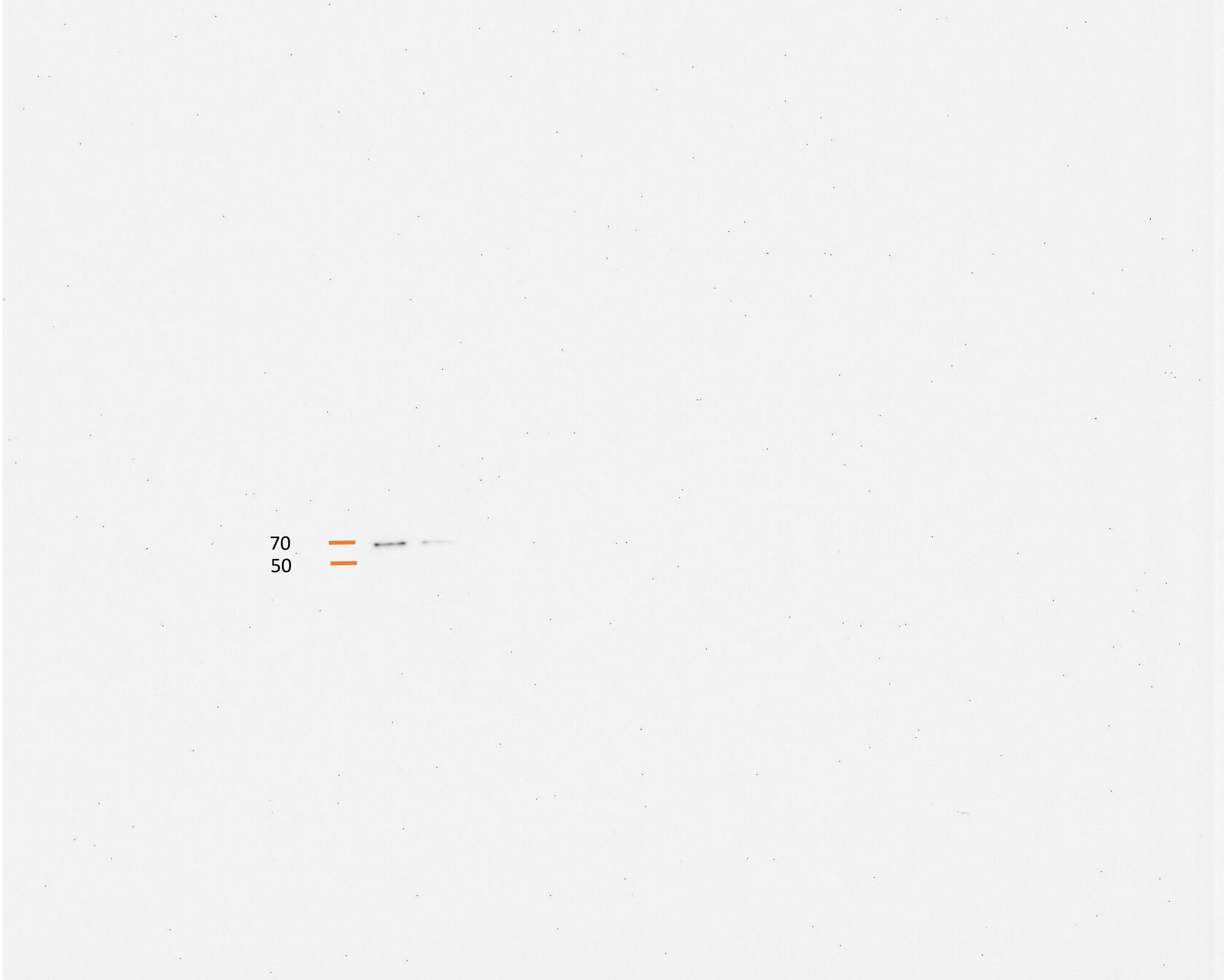

TFRC

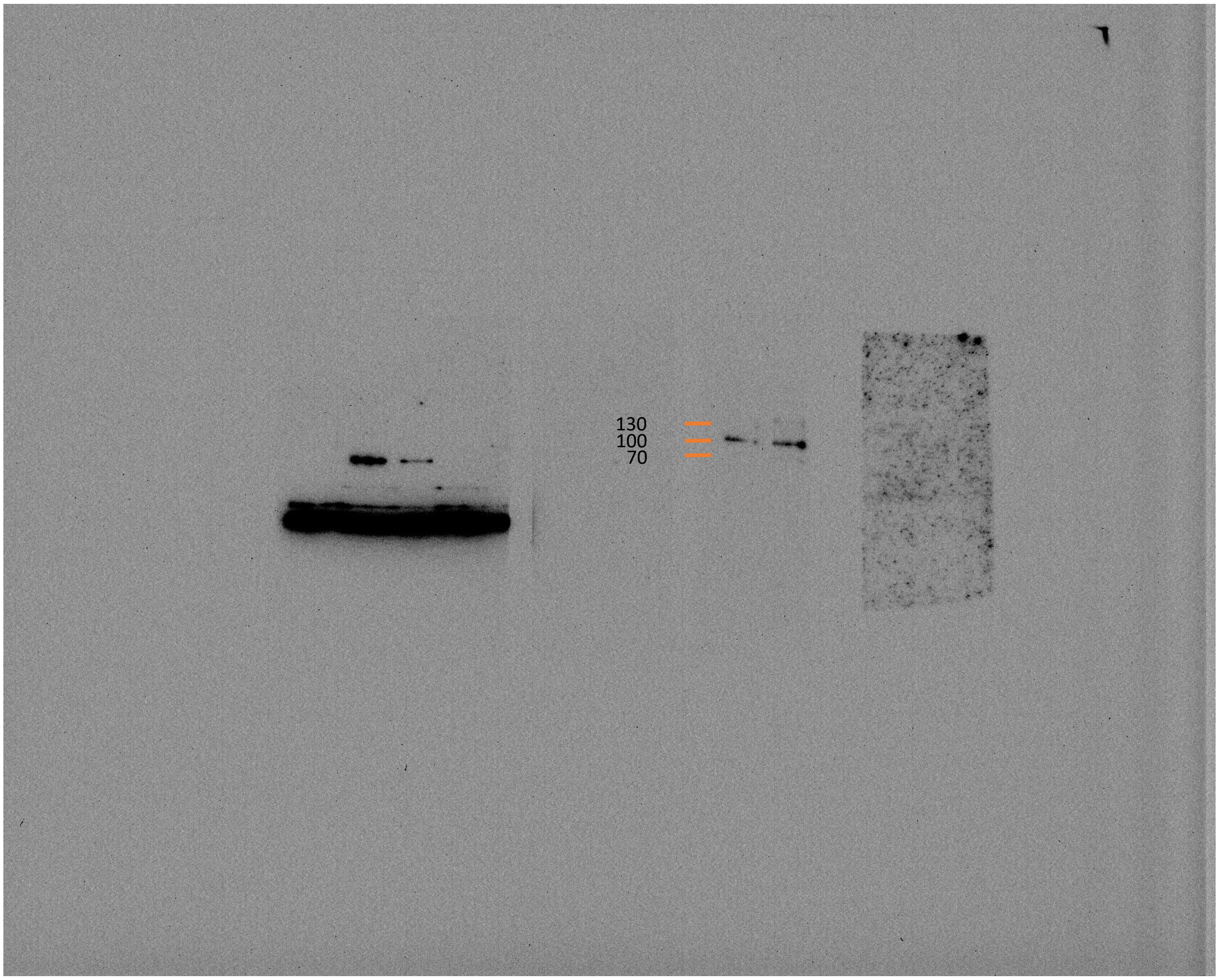

HMOX1

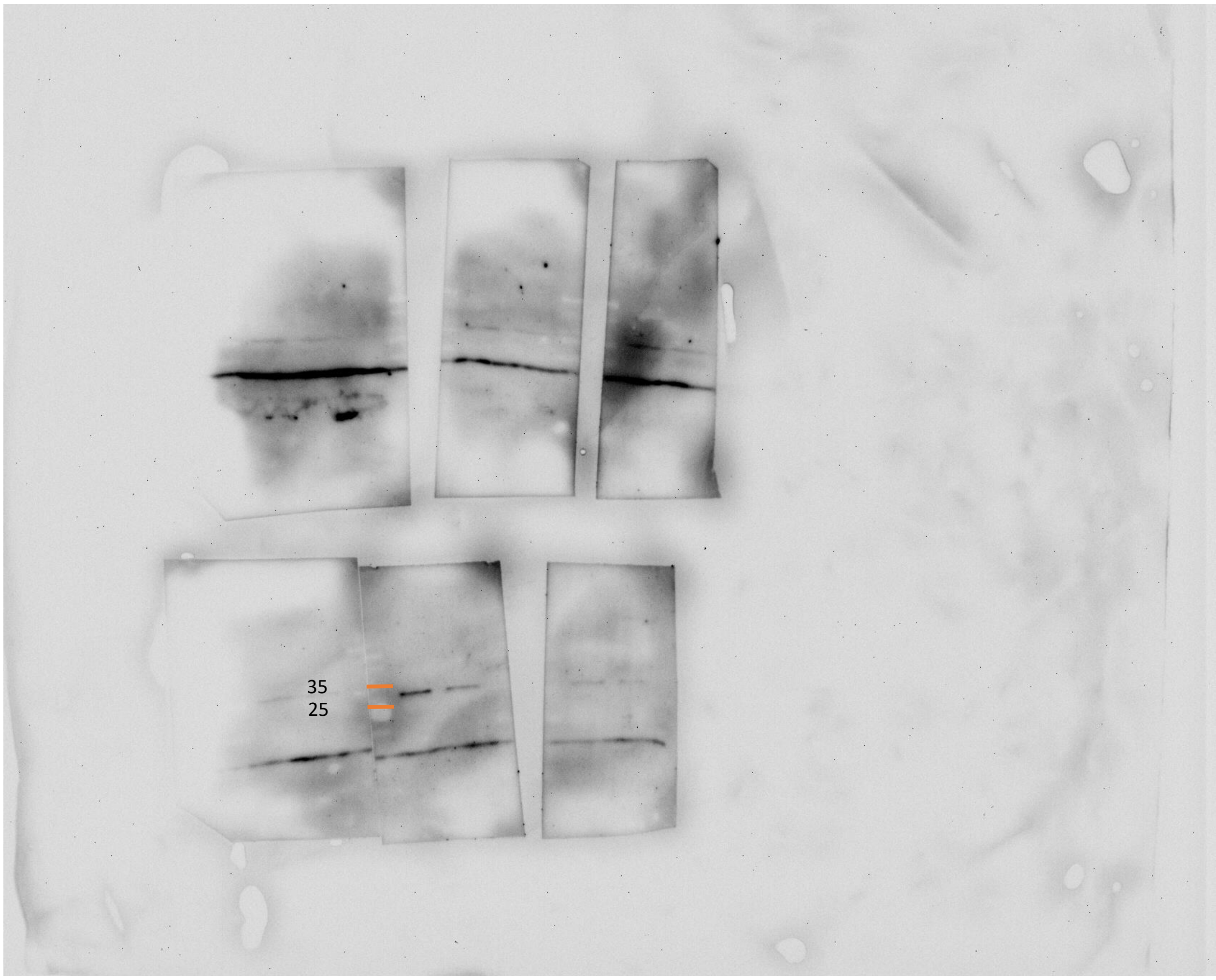

For quantification

PTGS2

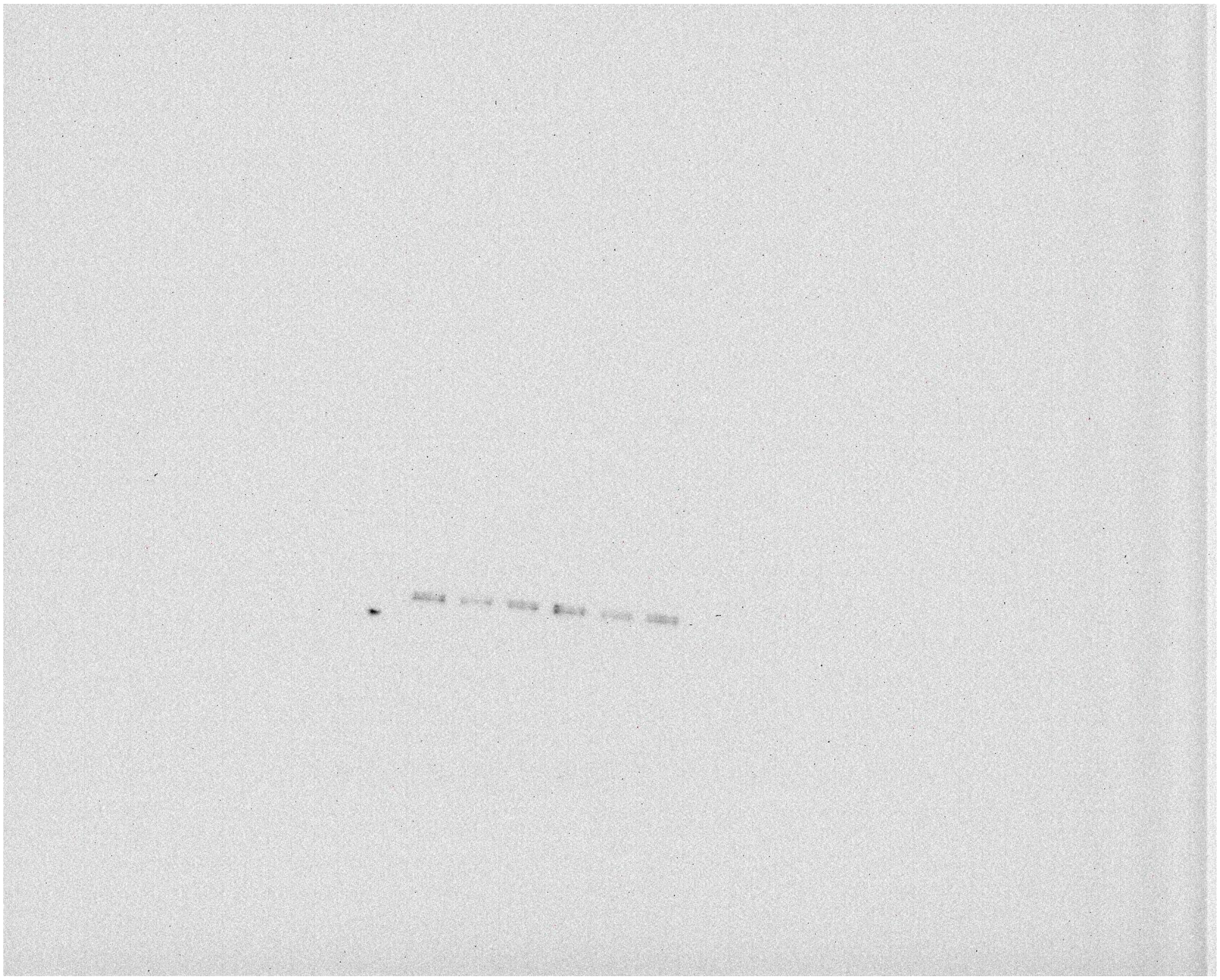

Total protein first 3 lanes control, last 3 OE

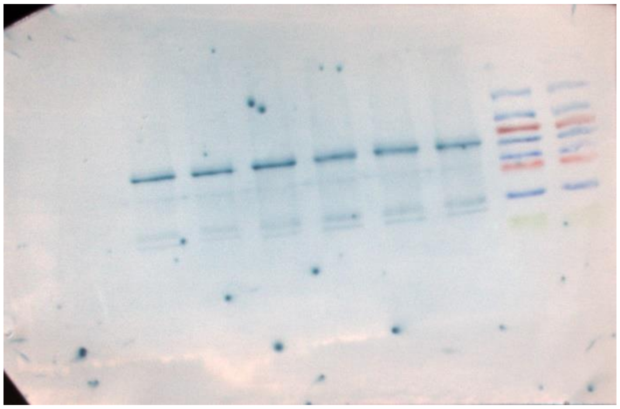

FABP5 total protein first 3 lanes OE, last 3 control

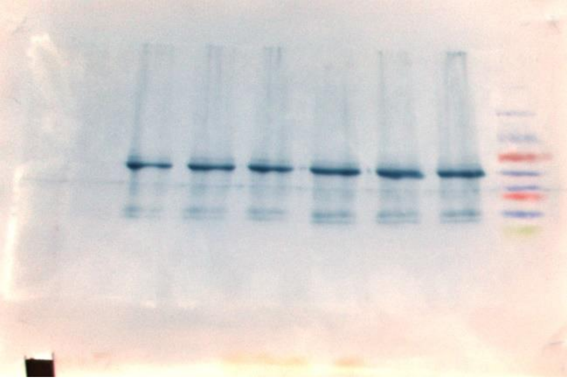

FABP5 (Fig 3 A)

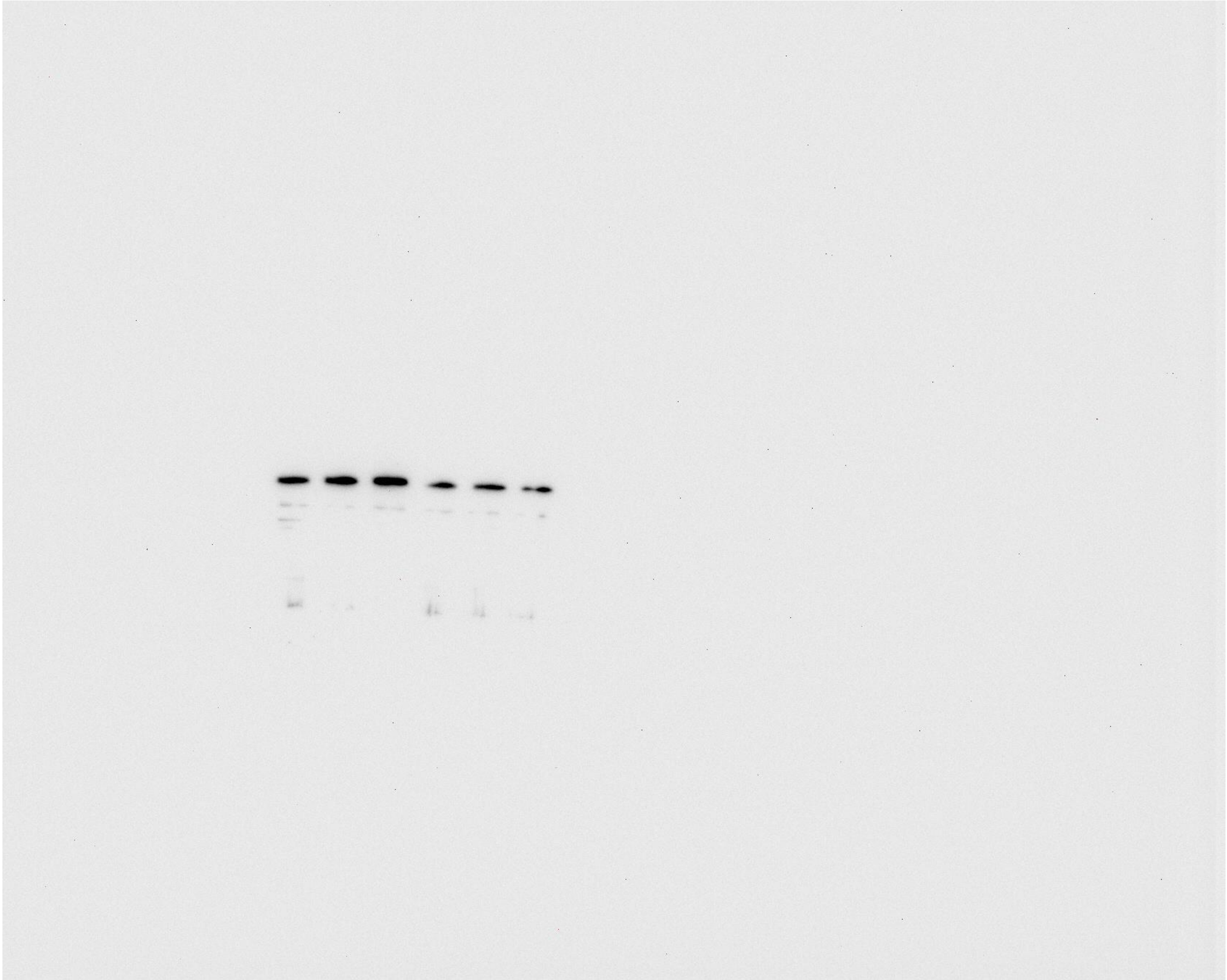

HMOX1 total protein first 3 lanes OE, last 3 control

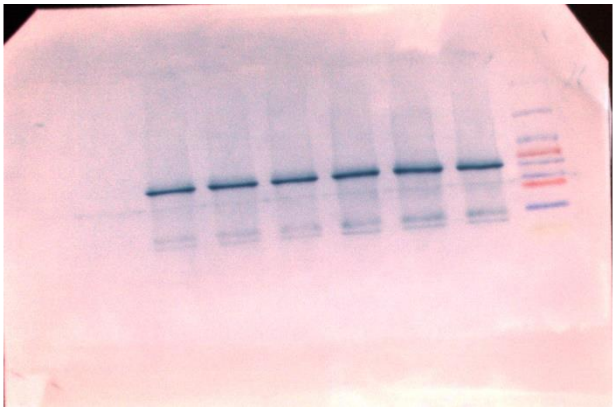

HMOX1

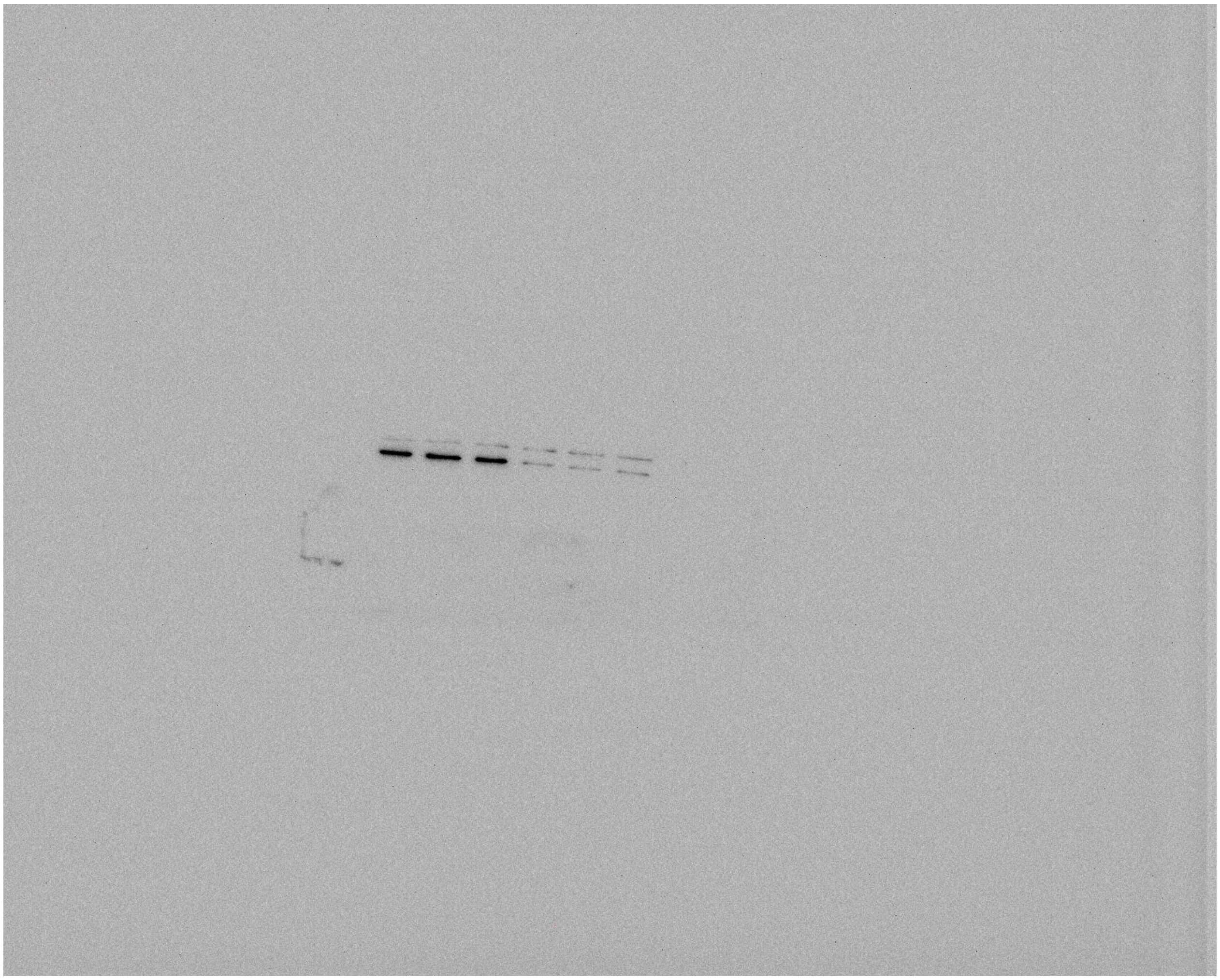

4HNE total protein first 3 lanes control, last 3 OE

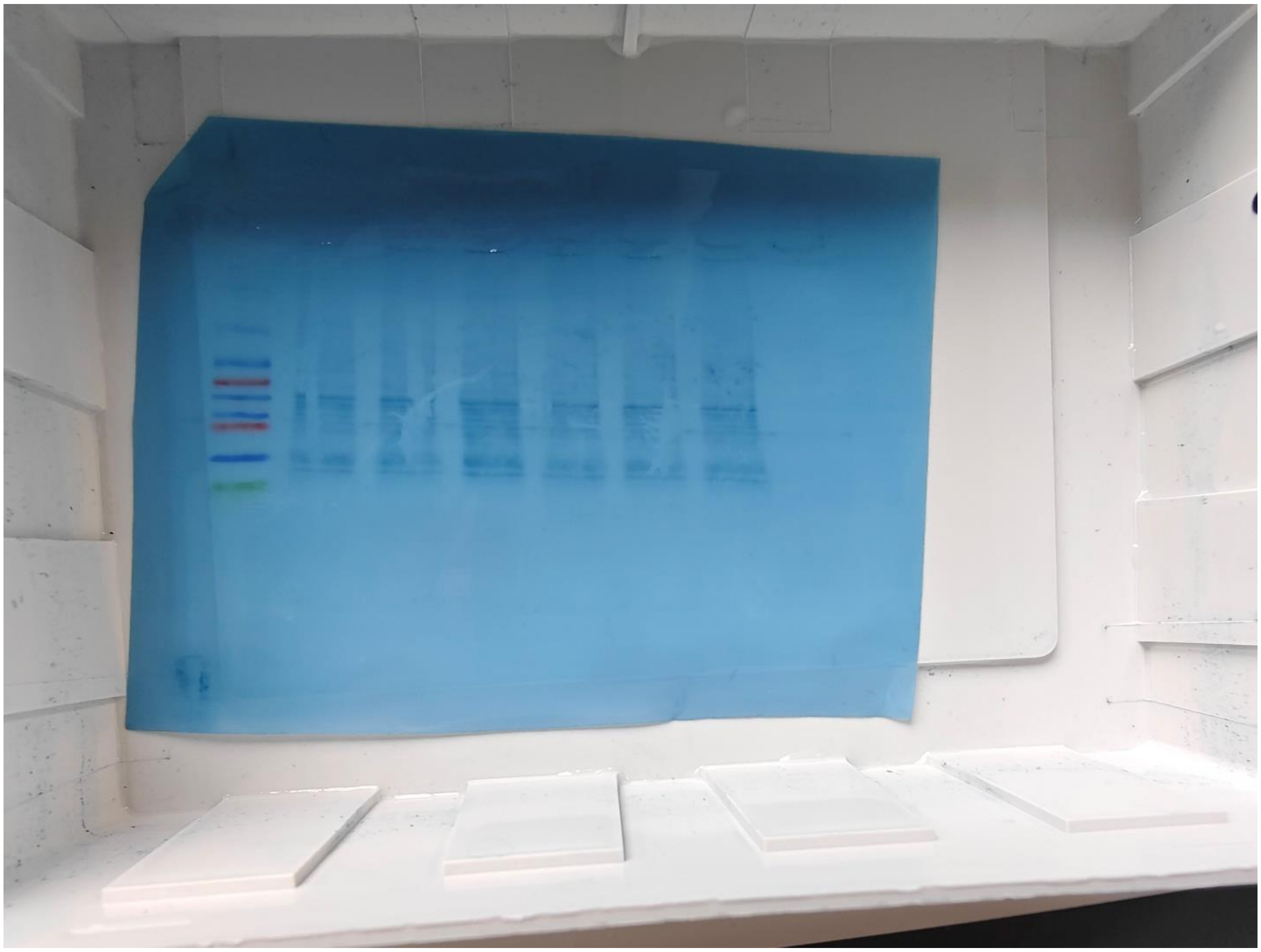

4HNE

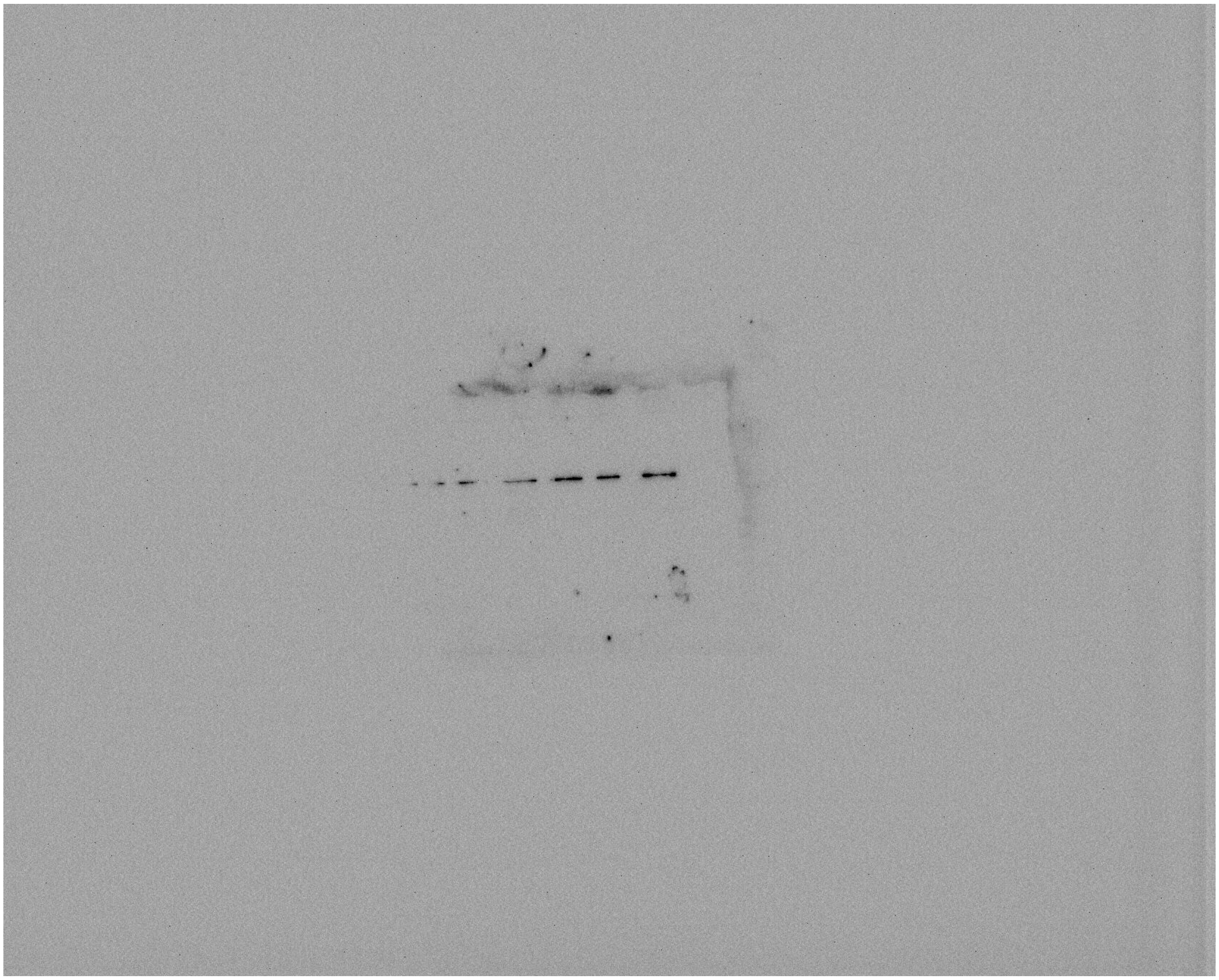

TRFC total protein first 3 lanes OE, last 3 control

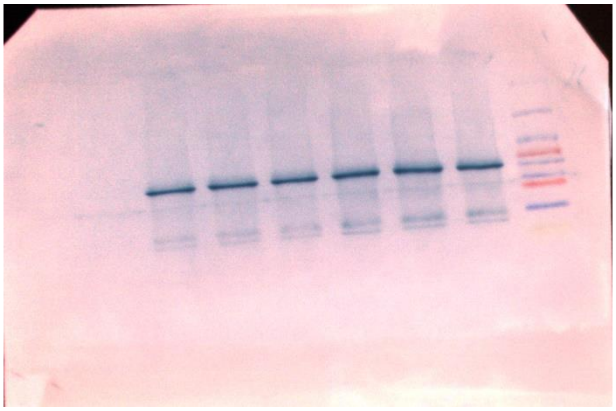

TFRC

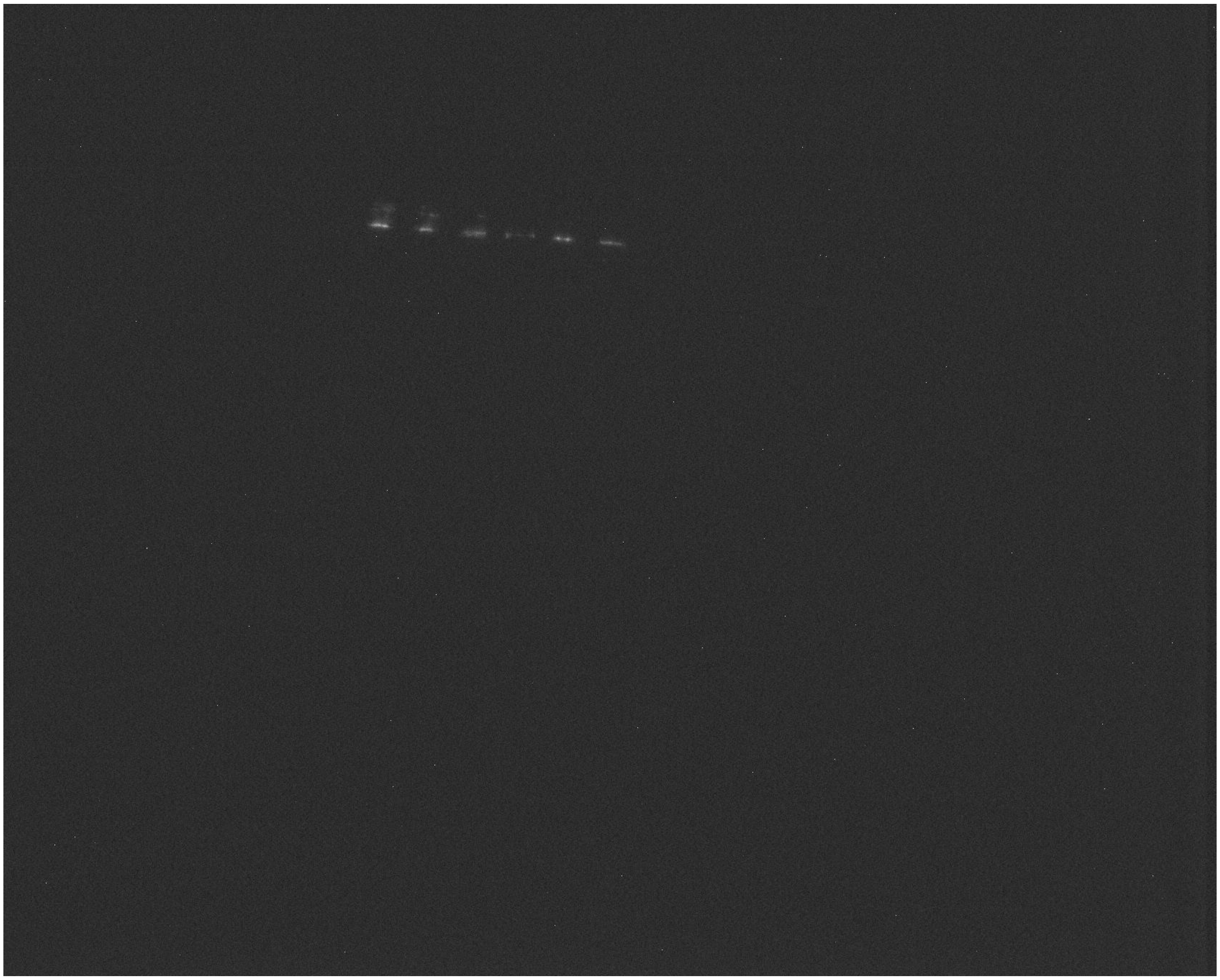

Fig 4C actin

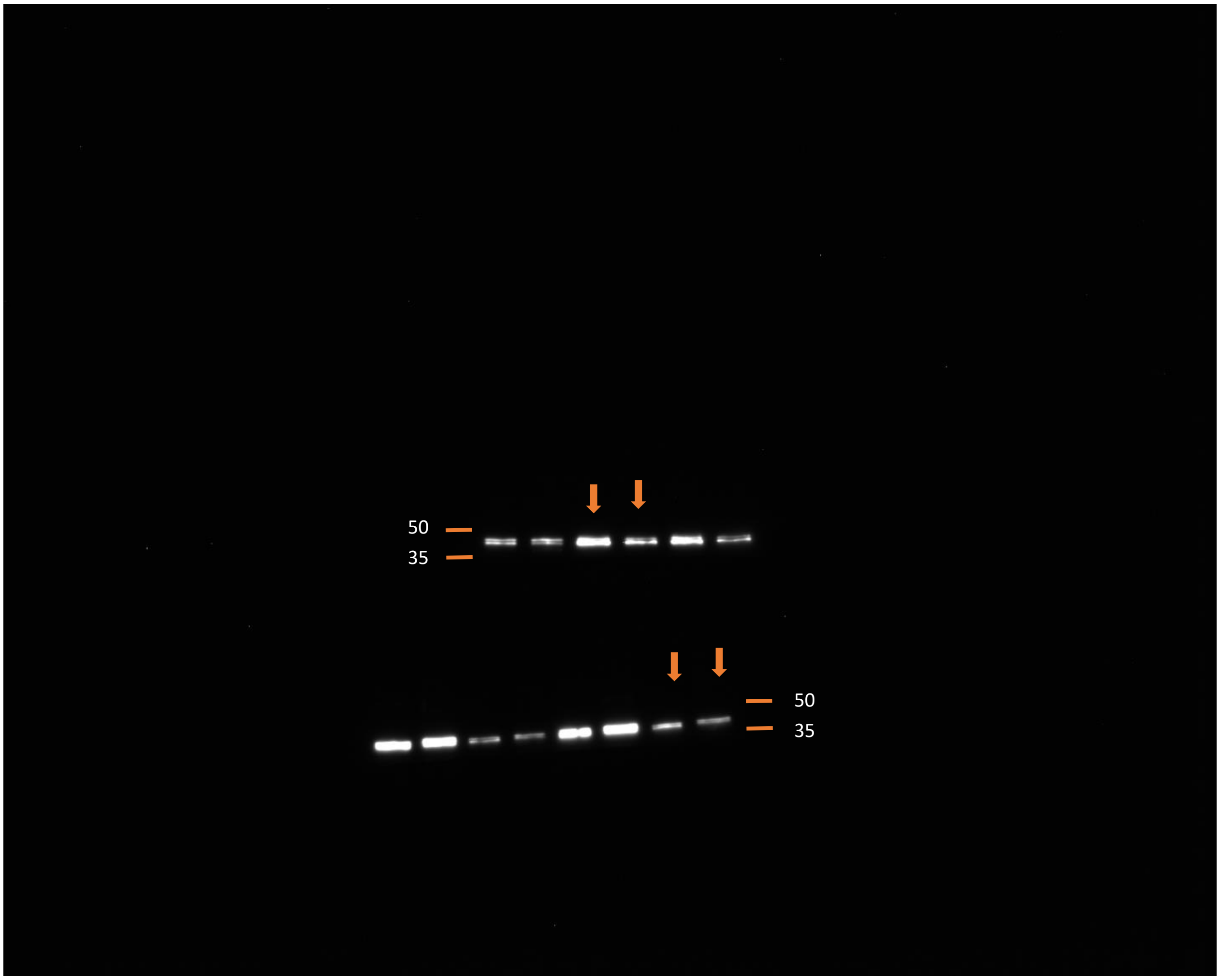

PRNP OE RAC3

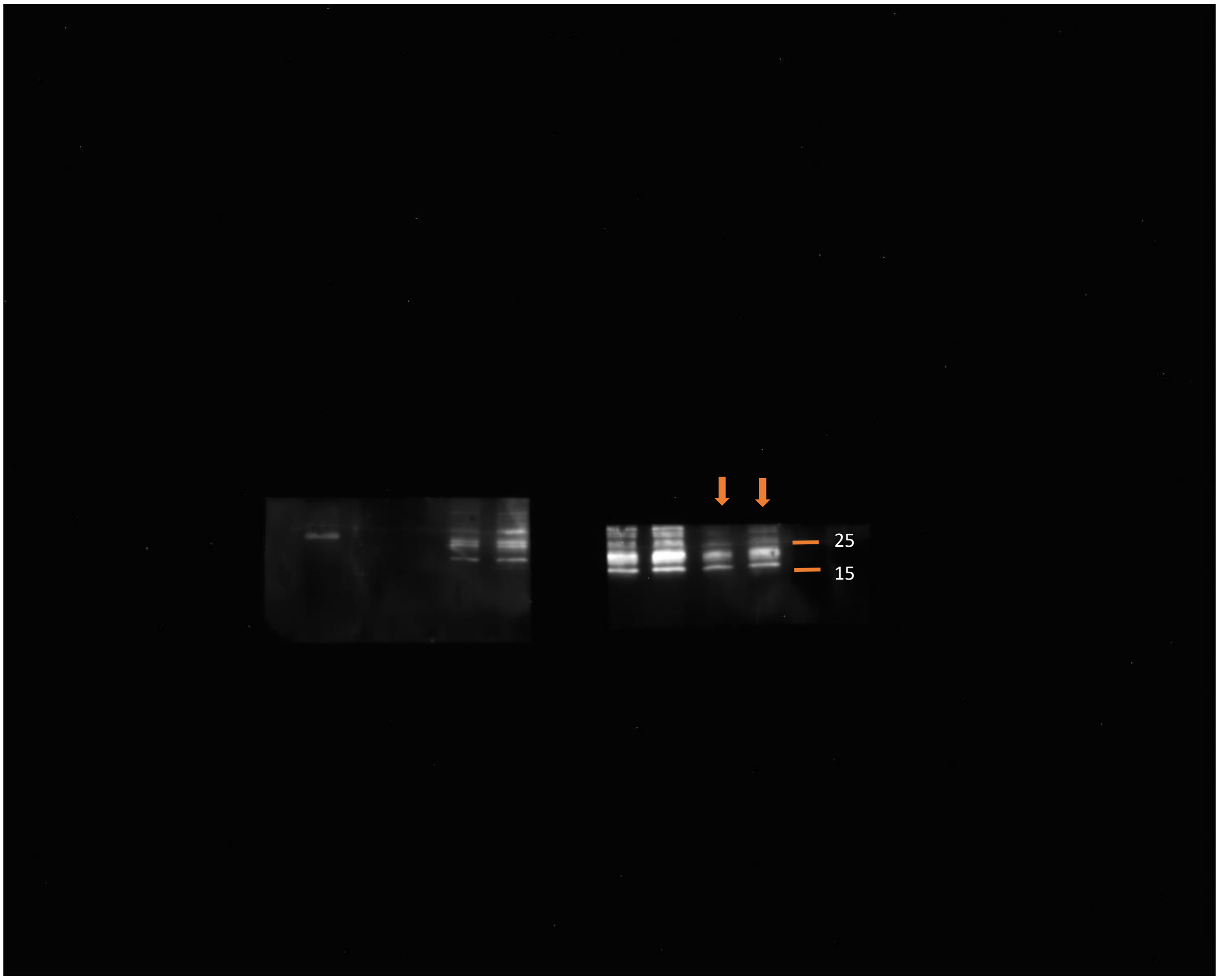

RAC3 OE PRNP

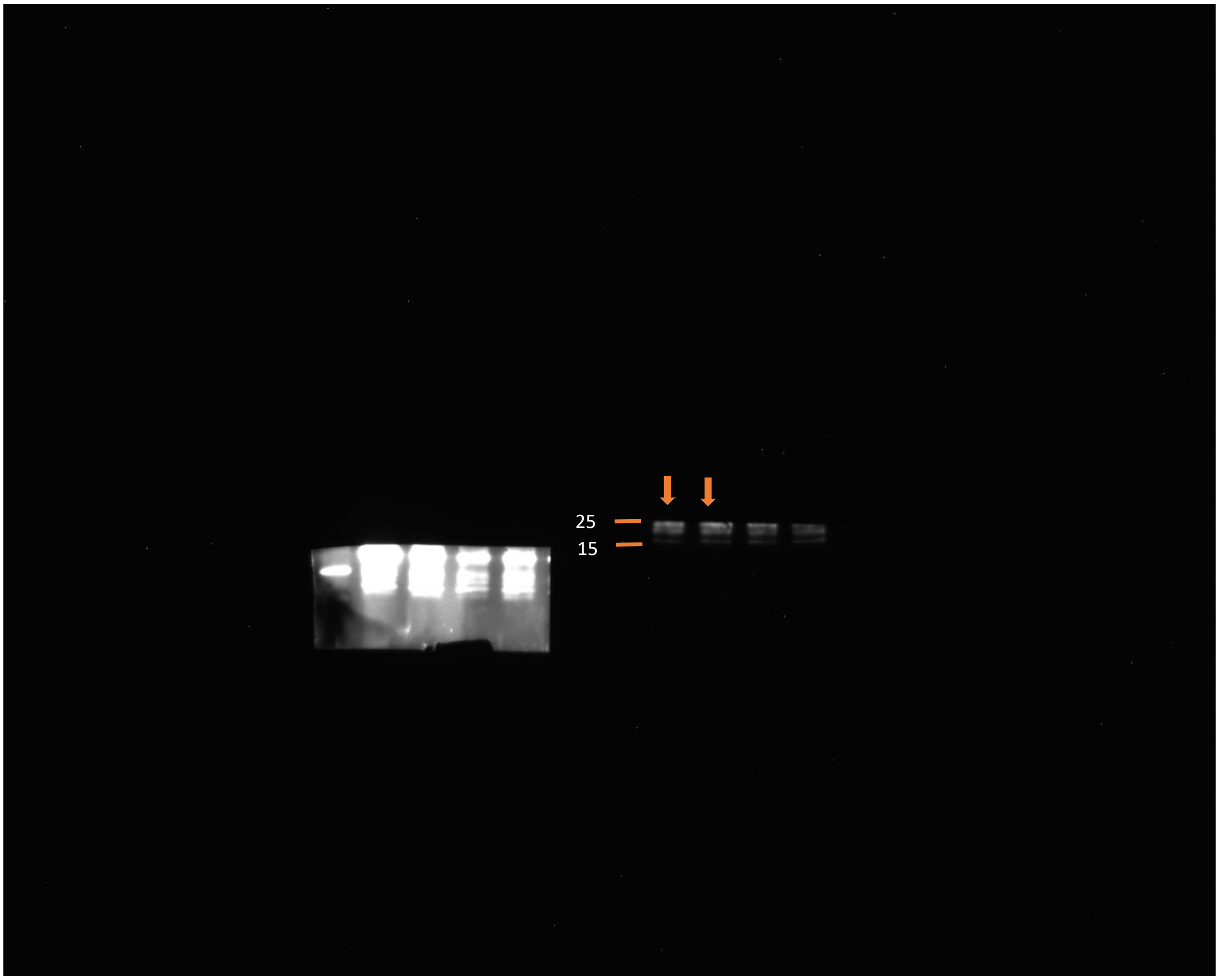

Fig 5B actin

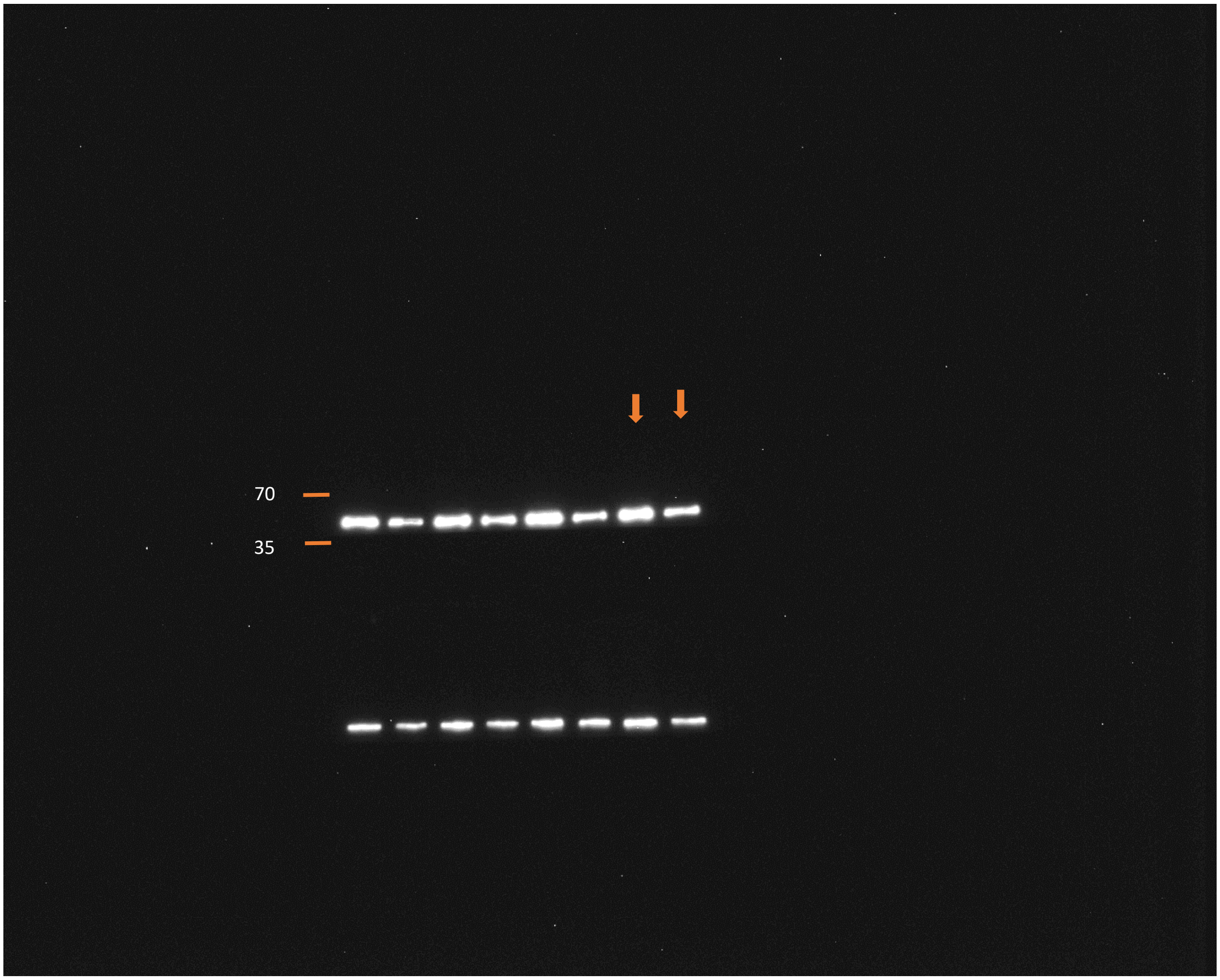

RAC3

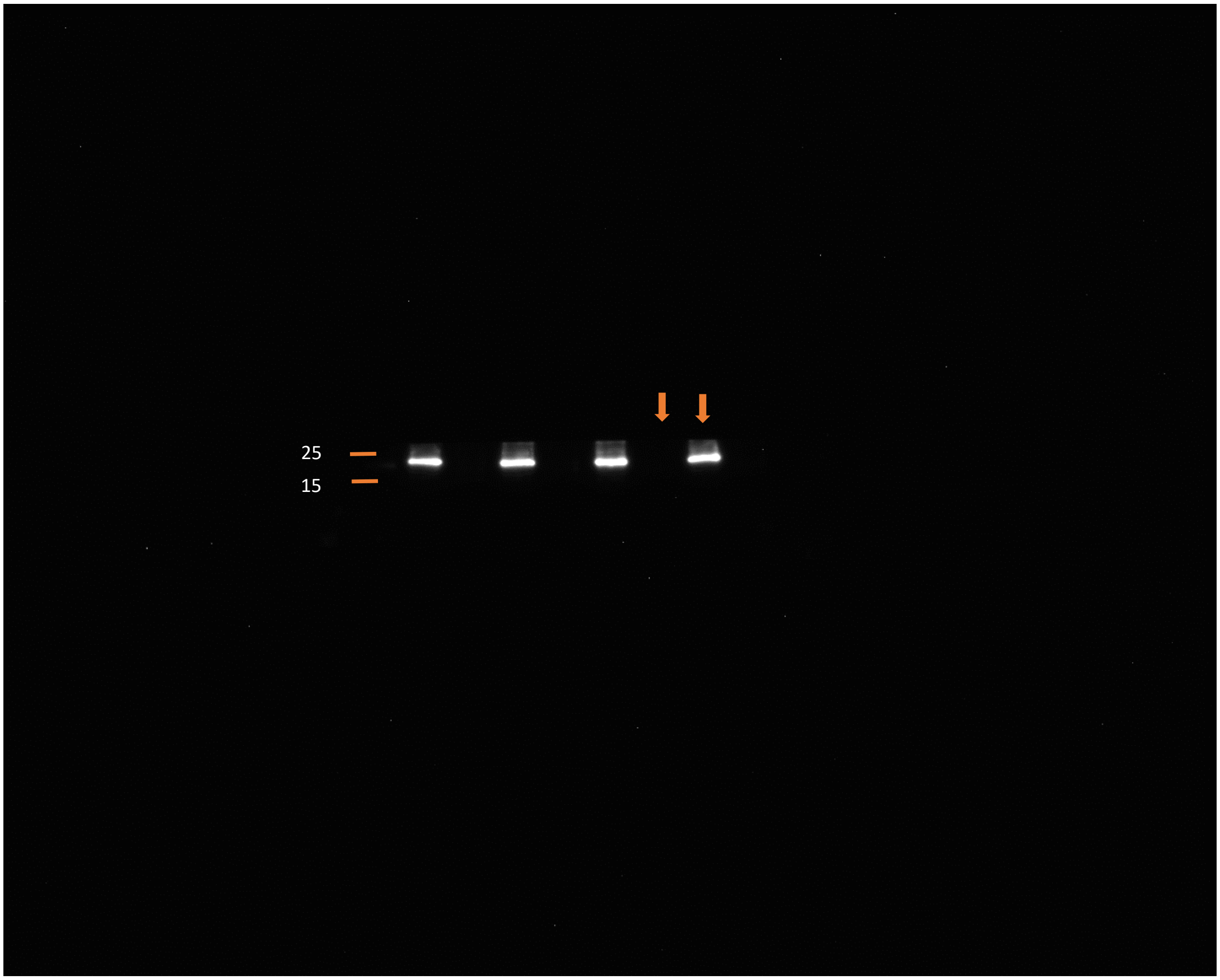

Supplement: Supplementary file 4 — Source data [file 41467_2025_60793_MOESM4_ESM.zip › All main figures wb raw membrane combine.pdf]
